# Supplementary material for: Comparative chromosome painting in Spizaetus tyrannus and Gallus gallus with the use of macro- and microchromosome probes
Source: PLoS One. 2021 Nov 18;16(11):e0259905. doi: 10.1371/journal.pone.0259905 (PMC8601422; doi:10.1371/journal.pone.0259905)

PLOS ONE

Comparative Chromosome Painting in the Black-Hawk-Eagle (Spizaetus tyrannus) and Gallus gallus with the use of Macro and Microchromosome Probes.

--Manuscript Draft--

**Manuscript Number:**	PONE-D-21-20639
**Article Type:**	Research Article
**Full Title:**	Comparative Chromosome Painting in the Black-Hawk-Eagle (Spizaetus tyrannus) and Gallus gallus with the use of Macro and Microchromosome Probes.
**Short Title:**	Compara[illegible] Chromosome Painting in the Black-Hawk-Eagle (Spizaetus tyrannus) and Gallus g[illegible]
**Corresponding Author:**	Edivaldo Herculano Corrêa de Oliveira<br>Universidade Federal do Pará<br>Belém, Para BRAZIL
**Keywords:**	Aquilinae; Chromosome painting; BACs; Microchromosomes

**Abstract:**	Accipitriformes are characterized by a large karyotypic reorganization, which resulted in complements with low diploid numbers, and a smaller number of microchromosomal pairs when compared to the karyotypes of most other birds, with diploid numbers around 2n=80 and many pairs of microchromosomes. Among Accipitriformes, the Accipitridae family is the most diverse and is currently divided into 14 subfamilies, including the subfamily Aquilinae, composed of medium to large sized species, including the Black-Hawk-Eagle (Spizaetus tyrannus - STY) found in South America. Here, we present the chromosome homologies between this species and the chicken using comparative chromosome painting. Our results revealed that at least 29 fission-fusion events were observed in the STY karyotype, based on homology with G[illegible] The majority of fissions occured in segments homologous to GGA1-GGA5, wherea[illegible]e majority of the GGA microchromosomes remained conserved and fused to other chromosomal elements in STY. When compared to the Japanese-Mountain-Eagle (Nisaetus nipalensis orientali[illegible] the only close relative of S. tyrannus analyzed by comparative chromosome p[illegible]ng so far, we observed that there are many similarities between these species, including numerous fissions of the first five pairs homologous to GGA with only one less in STY (21 events against 22 in NNI), and three fusions involving homologues of GGA1-GGA9 chromosomes and microchromosomes, but with breakpoints that are not shared between these two species.

**Order of Authors:**	Carlos A Carvalho
	Ivanete O Furo
	Patricia C.M. O´Brien
	Jorge Pereira
	Rebeca E O´Connor
	Darren Griffin
	Malcolm Ferguson-Smith
	Edivaldo Herculano Corrêa de Oliveira

**Additional Information:**	
**Question**	**Response**
**Financial Disclosure**<br><br>Enter a financial disclosure statement that describes the sources of funding for the work included in this submission. Review the submission guidelines for detailed	This research was partially funded by a grant to EHCO from CNPq (307382/2019-2) and to MAFS from the Wellcome Trust in support of the Cambridge Resource Centre for Comparative Genomics and by the Biotechnology and Biological Sciences Research Council (BB/K008161/1) to the University of Kent.

Powered by Editorial Manager® and ProduXion Manager® from Aries Systems Corporation

requirements. View published research articles from *PLOS ONE* for specific examples.<br><br>This statement is required for submission and **will appear in the published article** if the submission is accepted. Please make sure it is accurate.<br><br>**Unfunded studies**<br>Enter: *The author(s) received no specific funding for this work.*<br><br>**Funded studies**<br>Enter a statement with the following details:<br>• Initials of the authors who received each award<br>• Grant numbers awarded to each author<br>• The full name of each funder<br>• URL of each funder website<br>• Did the sponsors or funders play any role in the study design, data collection and analysis, decision to publish, or preparation of the manuscript?<br>• **NO** - Include this sentence at the end of your statement: *The funders had no role in study design, data collection and analysis, decision to publish, or preparation of the manuscript.*<br>• **YES** - Specify the role(s) played.<br><br>* typeset	
**Competing Interests**<br><br>Use the instructions below to enter a competing interest statement for this submission. On behalf of all authors, disclose any competing interests that could be perceived to bias this work—acknowledging all financial support and any other relevant financial or non-financial competing interests.<br><br>This statement is required for submission and **will appear in the published article** if the submission is accepted. Please make sure it is accurate and that any funding sources listed in your Funding Information later in the submission form are also declared in your Financial Disclosure	The authors have declared that no competing interests exist.

statement.<br><br>View published research articles from *PLOS ONE* for specific examples.<br><br>**NO authors have competing interests**<br><br>Enter: *The authors have declared that no competing interests exist.*<br><br>**Authors with competing interests**<br><br>Enter competing interest details beginning with this statement:<br><br>*I have read the journal's policy and the authors of this manuscript have the following competing interests: [insert competing interests here]*<br><br>* typeset	
**Ethics Statement**<br><br>Enter an ethics statement for this submission. This statement is required if the study involved:<br><br>• Human participants<br>• Human specimens or tissue<br>• Vertebrate animals or cephalopods<br>• Vertebrate embryos or tissues<br>• Field research<br><br>Write "N/A" if the submission does not require an ethics statement.<br><br>General guidance is provided below. Consult the submission guidelines for detailed instructions. **Make sure that all information entered here is included in the Methods section of the manuscript.**	The experiments were carried out according to the ethical protocols approved by an ethics committee (CEUA—Federal University of Pará) under no. 170/2013 and SISBIO 68443-1.

**Format for specific study types**<br><br>**Human Subject Research (involving human participants and/or tissue)**<br>• Give the name of the institutional review board or ethics committee that approved the study<br>• Include the approval number and/or a statement indicating approval of this research<br>• Indicate the form of consent obtained (written/oral) or the reason that consent was not obtained (e.g. the data were analyzed anonymously)<br><br>**Animal Research (involving vertebrate animals, embryos or tissues)**<br>• Provide the name of the Institutional Animal Care and Use Committee (IACUC) or other relevant ethics board that reviewed the study protocol, and indicate whether they approved this research or granted a formal waiver of ethical approval<br>• Include an approval number if one was obtained<br>• If the study involved *non-human primates*, add *additional details* about animal welfare and steps taken to ameliorate suffering<br>• If anesthesia, euthanasia, or any kind of animal sacrifice is part of the study, include briefly which substances and/or methods were applied<br><br>**Field Research**<br><br>Include the following details if this study involves the collection of plant, animal, or other materials from a natural setting:<br>• Field permit number<br>• Name of the institution or relevant body that granted permission	
**Data Availability**<br><br>Authors are required to make all data underlying the findings described fully available, without restriction, and from the time of publication. PLOS allows rare exceptions to address legal and ethical concerns. See the PLOS Data Policy and FAQ for detailed information.	Yes - all data are fully available without restriction

A Data Availability Statement describing where the data can be found is required at submission. Your answers to this question constitute the Data Availability Statement and **will be published in the article**, if accepted.<br><br>**Important:** Stating 'data available on request from the author' is not sufficient. If your data are only available upon request, select 'No' for the first question and explain your exceptional situation in the text box.<br><br>Do the authors confirm that all data underlying the findings described in their manuscript are fully available without restriction?	
**Describe where the data may be found in full sentences. If you are copying our sample text, replace any instances of XXX with the appropriate details.**<br><br>• If the data are **held or will be held in a public repository**, include URLs, accession numbers or DOIs. If this information will only be available after acceptance, indicate this by ticking the box below. For example: *All XXX files are available from the XXX database (accession number(s) XXX, XXX.*).<br>• If the data are all contained **within the manuscript and/or Supporting Information files**, enter the following: *All relevant data are within the manuscript and its Supporting Information files.*<br>• If neither of these applies but you are able to provide **details of access elsewhere**, with or without limitations, please do so. For example:<br><br>*Data cannot be shared publicly because of [XXX]. Data are available from the XXX Institutional Data Access / Ethics Committee (contact via XXX) for researchers who meet the criteria for access to confidential data.*<br><br>*The data underlying the results presented in the study are available from (include the name of the third party*	All relevant data are within the manuscript and its Supporting Information files.

Powered by Editorial Manager® and ProduXion Manager® from Aries Systems Corporation

and contact information or URL).<br>• This text is appropriate if the data are owned by a third party and authors do not have permission to share the data.<br><br>* typeset	
Additional data availability information:	Tick here if your circumstances are not covered by the questions above and you need the journal’s help to make your data available.

Manuscript

Click here to access/download;Manuscript;Carvalho et al. Manuscript.docx

Comparative Chromosome Painting in the Black-Hawk-Eagle (*Spizaetus tyrannus*) and *Gallus gallus* with the use of Macro and Microchromosome Probes.

Carlos Augusto L Carvalho[1], Ivanete de Oliveira Furo[1, 2, 3], Patricia C. M. O'Brien[3], Jorge Pereira J[4], Rebeca E. O'Connor[5], Darren Griffin[5], Malcolm A. Ferguson-Smith MA[3], Edivaldo Herculano C. de Oliveira[2,6] *

1 Programa de Pós-Graduação em Genética e Biologia Molecular, Universidade Federal do Pará, Belém, Pará 66075-110, Brazil

2 Laboratório de Cultura de Tecidos e Citogenética, SAMAM, Instituto Evandro Chagas, Ananindeua, Pará 67030-000, Brazil

3 Cambridge Resource Centre for Comparative Genomics, Cambridge CB3 0ES, UK

4 4 Animal and Veterinary Research Center, Universidade de Trá-os-Montes e Alto douro, Vila Real, Portugal

5 School of Biosciences, University of Kent, Canterbury CT2 7NJ, UK

6 Faculdade de Ciências Naturais, Instituto de Ciências Exatas e Naturais, Universidade Federal do Pará, Belém, Pará 66075-110, Brazil

Corresponding Author:

***Dr. Edivaldo Herculano C. de Oliveira**

ehco@ufpa.br, edivaldooliveira@iec.gov.br

Abstract

Accipitriformes are characterized by a large karyotypic reorganization, which resulted in complements with low diploid numbers, and a smaller number of microchromosomal pairs when compared to the karyotypes of most other birds, with diploid numbers around 2n=80 and many pairs of microchromosomes. Among Accipitriformes, the Accipitridae family is the most diverse and is currently divided into 14 subfamilies, including the subfamily Aquilinae, composed of medium to large sized species, including the Black-Hawk-Eagle (*Spizaetus tyrannus* - STY) found in South America. Here, we present the chromosome homologies between this species and the chicken using comparative chromosome painting. Our results revealed that at least 29 fission-fusion events were observed in the STY karyotype, based on homology with GGA. The majority of fissions occured in segments homologous to GGA1-GGA5, whereas the majority of the GGA microchromosomes remained conserved and fused to other chromosomal elements in STY. When compared to the Japanese-Mountain-Eagle (*Nisaetus nipalensis orientalis*), the only close relative of *S. tyrannus* analyzed by comparative chromosome painting so far, we observed that there are many similarities between these species, including numerous fissions of the first five pairs homologous to GGA with only one less in STY (21 events against 22 in NNI), and three fusions involving homologues of GGA1-GGA9 chromosomes and microchromosomes, but with breakpoints that are not shared between these two species.

Key Words: Aquilinae, Chromosome painting, BACs, Microchromosomes

Introduction

Excluding the New World vultures (Cathartidae), which show similar karyotypes to the putative avian ancestral karyotype [1], species belonging to the Order Accipitriformes present with an unusual chromosomal diversity. They have smaller diploid numbers, 2n = 54-68, and a reduction of microchromosomes to between 4 and 8 pairs [2-4]. According to comparative chromosome painting studies using whole chromosome paints of the chicken (*Gallus gallus* – GGA), this large reorganization included several fissions in the macrochromosome pairs homologous to GGA1-GGA5, while microchromosomes were largely involved in fusion events [5-10].

Due to difficulties involving the isolation of individual microchromosome pairs by flow cytometry for specific probe production, most data have been obtained using pools of microchromosomes, i.e., chromosome paints that recognize more than one pair. Therefore, improved identification of chromosome pairs involved in rearrangements is a priority if we are to achieve a more definitive analysis and identify synapomorphies based on chromosome characters [11-12].

The Black-Hawk-Eagle (*Spizaetus tyrannus*) belongs to the subfamily Aquilinae (Accipitriformes, Accipitridae), and is found in South and Central American from southern Mexico down to Argentina (Sick, 1997; Mikich & Bernils, 2004). The only chromosomal analysis of *S. tyrannus* to date was based on conventional staining, revealing a karyotype within the Accipitridae standard, with 2n=68 and only four pairs of microchromosomes [1].

This study presents the cytogenetic mapping of a species of the genus *Spizaetus* by comparative painting. In addition to whole-chromosome paints of *Gallus gallus* (GGA), we used BAC probes from GGA clones that identified 11 individual pairs of microchromosomes.

Results

The karyotype of *Spizaetus tyrannus* presents a diploid number 2n=68, consisting of 21 meta or submetacentric pairs (pairs 1-4, 6-7, 9-10, 12, 14, 16-17, 19-22, 24-28 and the sex chromosomes), 7 acrocentric (pairs 5, 8, 11, 13, 15, 18 and 23), and 4 pairs of microchromosomes (pairs 29-33). The Z chromosome is a large metacentric, with size between pairs 3 or 4, while the W chromosome is an average submetacentric, similar in size to pairs 8 or 9 (Figure 1).

Comparative Chromosome painting

GGA probes used in the FISH experiments produced reproducible results. Hybridizations with chromosome-specific probes for the first ten pairs of GGA produced 22 signals, with the first five pairs producing multiple signals, ranging from 2 to 6 signals (Figure 2). For instance, GGA1 probe painted six distinct pairs in the STY karyotype (pairs 5, 6, 12, 14, 18, 25), while the probes GGA6-10 pairs showed only one signal each. Table 1 details the distribution of the signals produced by GGA whole specific probes in the karyotype of *Spizaetus tyrannus*.

Table 1- Results of hybridizations with *Gallus gallus* probes showing the homology between GGA probes in the karyotype of *Spizaetus tyrannus*.

Probe	STY Chromosomes	Probe	STY Chromosomes
**GGA1**	(5, 6, 12, 14, 18, 25)	**GGA6**	9
**GGA2**	(1, 3q, 21)	**GGA7**	8
**GGA3**	(13, 16q, 19, 20)	**GGA8**	7
**GGA4**	(2, 17)	**GGA9**	11q
**GGA5**	(4, 15q)	**GGA10**	10q

A total of 19 out of 22 *Gallus gallus* BAC clones produced results. Both BACs from the GGA22 chromosome did not produce any detectable signal, as well as one of the BACs from GGA21. Among the 19 probes that gave good quality results, both proximal (BACp) and distal (BACd) referring to 8 pairs, were found in the same segment in the STY karyotype. However, BACs corresponding to GGA17 hybridized to two different pairs - BAC17p marked STY 9q, while BAC17d marked STY 24q. (Figure 3). All Chicken BACs and their respective homology in the karyotype of *Spizaetus tyrannus* are summarized in Table 2.

Table 2: Summary of the results of experiments using GGA BACs on the karyotype of *Spizaetus tyrannus* (*=BACs marking the same segment in STY karyotype; p=proximal region; d=distal region).

GGA	BAC ID	STY	GGA	BAC ID	STY
**17p**	CH261-113A7	9q	**23d**	CH261-90K11	23p*
**17d**	CH261-42P16	24q	**24p**	CH261-103F4	15p*
**18p**	CH261-60N6	19p*	**24d**	CH261-65O4	15p*
**18d**	CH261-72B18	19p*	**25p**	CH261-59C21	20p*
**19p**	CH261-10F1	13p*	**25d**	CH261-127K7	20p*
**19d**	CH261-50H12	13p*	**26p**	CH261-186M13	27p*
**21p**	CH261-83I20	No signal	**26d**	CH261-170L23	27p*
**21d**	CH261-122K8	4p	**27p**	CH261-66M16	16p*
**22p**	CH261-40J9	No signal	**27d**	CH261-28L10	16p*
**22d**	CH261-18G17	No signal	**28p**	CH261-64A15	11p*
**23p**	CH261-191G17	23p*	**28d**	CH261-72A10	11p*

Homologies obtained both by whole chromosome painting and BAC probes are shown in figure 4.

Discussion

The karyotype of *Spizaetus tyrannus* obtained herein presented 2n=68, confirming data from a previous report [1]. We report slight differences in chromosome morphology however, due to the higher number of biarmed pairs.

The results of comparative chromosome painting with whole chromosome probes of *Gallus gallus* showed a similar pattern to other birds of prey in the family Accipitridae, with a large reorganization of the syntenic groups homologous to the first five pairs of *Gallus*. That is, each probe (GGA1 - GGA5) corresponded to at least two distinct pairs (Figure 3). The most extreme examples are the fission of GGA1 into six pairs in STY, and GGA3 into three distinct pairs. These results are congruent with other birds of prey, considering that GGA1 can reveal syntenic segments in four pairs (*Gypaetus barbatus*) to seven pairs (*Nisaetus nipalensis orientalis* - NNI), while GGA3 is hybridized to four pairs in all species analyzed in this family. The exception is NNI where it hybridizes to only 2 pairs [10]. On the other hand, GGA6 - GGA10 are conserved syntenies, with only one signal for each pair.

All associations observed in the karyotype of STY based in its homology with *Gallus gallus* are represented in Figure 4. In general, 16 fissions and 13 fusions were detected, totalizing 29 rearrangements in the karyotype of STY when compared to *Gallus*, with fissions occurring mainly in relation to the first five pairs of macrochromosomes and fusions involving mainly the microchromosomes. In the microchromosomes, chicken BAC probes showed that their syntenies were not disrupted by fission events as probes for proximal and distal regions were found hybridizing to the same pair in STY. However, all the identified BAC signals showed that each GGA microchromosome was fused to a STY segment homologous to either a GGA macro or microchromosome. This indicates that chromosomal fusion led to the lower diploid number in STY and other

Accipitriformes. Despite the numerous fissions observed, it is important to note that not all GGA microchromosomes are represented by chicken BACs, and hence other fusions must have occurred in this species to maintain a 2n = 68.

The closest species to *Spizaetus tyrannus* with chromosome painting data is the Japanese-Eagle (*Nisaetus nipalensis orientalis*) [9]. Although geographically separated, they are morphologically similar, and until the last decade were classified as part of the same genus. Despite now being separated into distinct genera, molecular data support their close phylogenetic relationship [14]. Nevertheless, the comparative chromosome painting detects many differences. For instance, GGA1-9 probes produced signals in 21 pairs in STY, and 22 in NNI; the difference was due to an extra fission of GGA1 in NNI. Despite both species presenting three fusions involving the first nine pairs with microchromosomes (STY4, 7 and 9; NNI2, 4 and 9), none of them share the same GGA syntenic groups, and microchromosomes involved in NNI were not identified. Additionally, in both species GGA3 hybridizes to 4 pairs, however in STY all these segments are fused with microchromosomes (GGA18, 19, 24 and 25), whereas in NNI only one segment of GGA3 is fused with a microchromosome (unidentified pair) [9].

Another point relates to the difference in diploid number of *S. tyrannus* and *N. nipalensis* (2n = 66 in NNI and 2n = 68 in STY). This can be explained by a centric fusion of two pairs of microchromosomes (pairs not identified) in the NNI7 chromosome resulting in one pair of microchromosomes less in the karyotype of *N. nipalensis* [9]. These results show that they are morphologically similar species that until the last decade were part of the same genus [14].

Moreover, despite STY and NNI presenting some karyotypic similarities common to diurnal birds of prey such as recurrent breakpoints mainly in relation to the GGA1-GGA5 pairs [9,10], we did not identify any synapomorphic associations which could

represent ancestral characteristics for the Aquilinae [15,16]. Hence, while other subfamilies, such as Buteoninae and Harpiinae present well-established chromosomal signatures that allow the elaboration of their putative ancestral karyotypes [6], the available chromosome data indicate an absence of chromosomal signatures between STY and NNI, which can be explained by their significant geographic isolation, inhabiting opposite regions in the globe.

Conclusion

The present work is the first comparative chromosome mapping of a species in the genus *Spizaetus* and has revealed substantial karyotypic reorganization common to birds of prey of the family Accipitridae. Together with *Gallus* chromosome-specific probes for the larger pairs, chicken BACs were able to provide a more comprehensive result with additional information on the organization of the Black Hawk-Eagle karyotype. There are many similarities with the Japanese-Eagle, including numerous fissions of the first five pairs homologous to GGA with only one less in STY (21 events against 22 in NNI), and three fusions involving homologues of GGA1-GGA9 chromosomes and microchromosomes, but with breakpoints that are not shared between these two species. For a broader analysis at the phylogenetic level, it would be necessary to have comparative mapping of other species of the genus *Spizaetus* so that an ancestral karyotype of this genus could be traced. Furthermore, the new set of chromosome probes of *S.tyrannus* should be helpful in further studies of the phylogenetic relationships in Accipitriformes.

Methods

Samples and Chromosome Preparations

The experiments followed the standards approved by the Ethics Committee for the Use of Animals in Research (CEPAE-UFPA under number 170-13). We performed fibroblast cultures from skin biopsies and feather pulp of *Spizaetus tyrannus* (STY) obtained from two female individuals maintained in Zoos, following the protocol of Sasaki et al. [13] with modifications. After tissue cleavage in Petri dishes, the samples were incubated with 1% type 1 collagenase (GIBCO) for 1 hour at 37°C for tissue dissociation. Metaphase chromosomes were obtained after incubation for one hour with Colcemid (0.05 µg/mL), hypotonic solution (KCl at 0.075 M) for 30 minutes and fixation in methanol/acetic acid (3:1). Karyotype analysis was performed using conventional staining with 5% Giemsa in 0.07 M phosphate buffer (pH 6.8) for 5 minutes, slides were analyzed using a 100× objective (Leica, CO, USA) and GenASIs software (ADS Biotec, Omaha, NE, USA).

GGA Probes and FISH Experiments

Two types of *Gallus* probes were used: whole-chromosome-specific probes of the first 10 pairs, and chromosome-specific BAC probes from 11 microchromosome pairs. Whole chromosome paints were developed and provided by the Cambridge Resource Center for Comparative Genomics (Cambridge, UK) using the Fluorescent Activated Cell Sorting (FACS) technique and labeled with biotin, fluorescein and/or digoxigenin (Roche Diagnostics, Mannheim, Germany), and detected with the addition of avidin-Cy3 (or Cy5) or anti-digoxigenin-rhodamin (Vector Laboratories, Burlingame, CA, USA). BAC clones ranged from 150,000 kb to 210,000 kb in size were selected from the CHORI-261

Chicken BAC library (Children's Hospital Oakland Research Institute, Oakland-USA), corresponding to sequences from the proximal and distal regions of the microchromosomes (each pair represented by two BACs, in a total of 22 BACs covering 11 chromosome pairs). Clones were produced following the protocol of the mini prep kit (Qiagen, Hilden, Germany) and labelled directly by FTIC (green) or Texas Red (red) through Nick Translation (Roche, Mannheim, Germany).

Hybridization experiments followed standard procedures (de Oliveira et al., 2010; O´Connor et al., 2018). Slides were analyzed with an Olympus BX-61 epifluorescence microscope equipped with a cooled CCD camera and appropriate filters. Images were captured using SmartCapture3 (Digital Scientific UK).

Declarations

Ethics Approval: The experiments were carried out according to the ethical protocols approved by an ethics committee (CEUA—Federal University of Pará) under no. 170/2013 and SISBIO 68443-1.

Availability of Data and Materials: All data generated or analyzed during this study are included in this published article

Author Contributions: Conceptualization, C.C.A.L, I.O.F. and E.H.C.O.; Data curation and formal analysis, C.C.A.L., I.O.F., J.P. and E.H.C.O.; Investigation, C.C.A.L., I.O.F., Methodology, I.O.F., P.C.M.O., and J.P.; Project administration, E.H.C.O.; Funding acquisition, M.A.F.S., D.K.G. and E.H.C.O.; Validation, C.C.A.L., I.O.F. and E.H.C.O.; Writing (original draft), C.C.A.L., I.O.F. and E.H.C.O.; Writing (review and editing), D.K.G., P.C.M.O. and M.A.F.S.

Funding: This research was partially funded by a grant to EHCO from CNPq (307382/2019-2) and to MAFS from the Wellcome Trust in support of the Cambridge Resource Centre for Comparative Genomics and by the Biotechnology and Biological Sciences Research Council (BB/K008161/1) to the University of Kent.

Acknowledgements: We would like to thank all the staff of the Laboratório de Cultura de Tecidos e Citogenética (SAMAM, IEC) and Cambridge Resource Centre for Comparative Genomics for their technical support.

Conflict of interest: The authors declare that they have no conflict of interest

References

1. Tagliarini, M. M.; Nagamachi, C. Y.; Pieczarka, J. C. & De Oliveira, E. H. C. Description of two new karyotypes and cytotaxonomic considerations on Falconiformes. Brazilian J Ornit, 2007; 15(2): 167-172.

2. De Boer, L. E. M. The somatic chromosomes of 16 species of Falconiformes (Aves) and the karyological relationships of the order. Genética, 1990; 46: 77.

3. Bed'Hom B. *Etude des Caryotypes Atypiques des Accipitridae (Aves, Falconiformes) par Cytogènètique Classique et Mole¤ culaire, et Modèlisation de leur Evolution*. PhD thesis, University of Tours, 1999.

4. Santos, L. P. & Gunski, R. J. Revisão de dados citogenéticos sobre a avifauna brasileira. Brazilian J Ornith, 2006; 14: 35-45.

5. de Oliveira, E. H. C.; Habermann, F.; Lacerda, O.; Sbalqueiro, I. J.; Wienberg, J. & Muller, E. S. Chromosome reshuffling in birds of prey: the karyotypes of the world's largest eagle (Harpy eagle, *Harpia harpyja*) compared to that of the chicken (*Gallus gallus*). Chromosoma, 2005; 114: 338-343.

6. de Oliveira, E. H. C.; Tagliarini M. M.; Rissino J. D.; Pieczarka, J. C.; Nagamachi, C. Y.; O´Brien, P. C. M. & Ferguson-Smith, M. A. Reciprocal chromosome painting between white hawk (*Leucopternis albicollis*) and chicken reveals extensive fusions and fissions during karyotype evolution of accipitridae (Aves, Falconiformes). Chromosome Res, 2010; 18: 349–355.

7. de Oliveira, E. H. C.; Tagliarini M. M.; Santos, M. S.; O´Brien, P. C. M. & Ferguson-Smith, M. A. Chromosome painting in three species of buteoninae: a cytogenetic signature reinforces the monophyly of south american species. Plos One, 2013; 8(7), 5–10.

8. Nanda I; Karl E; Volobouev V; Griffin DK; Schartl M; Schmid M. Extensive gross genomic rearrangements between chicken and Old World vultures (Falconiformes: Accipitridae). Cytogenet Genome Res, 2006; 112:286-295.

9. Nishida C, Ishijima J, Ishishita S, Yamada K, Griffin DK, Yamazaki T, et al. Karyotype reorganization with conserved genomic compartmentalization in dot-shaped microchromosomes in the Japanese mountain hawk-eagle (*Nisaetus nipalensis orientalis*, Accipitridae). Cytogenet Genome Res, 2013;141:284–94.

10. Nie, W.; O´Brien, P. C. M.; Fu, B.; Wang, J.; Su, W.; He, K.; Bed´Hom, B. et al. Multidirectional chromosome painting substantiates the occurrence of extensive genomic reshuffling within Accipitriformes. BMC Evolutionary Biology, 2015; 15:205.

11. Crooijmans, R.; Vrebalov, J.; Dijkhof, R; Van Der Poel, J. & Groenen, M. Two-dimensional screening of the Wageningen chicken BAC library. Mammalian Genome, 2000; 11(5): 360-363.

12. Lithgow, P. E.; O´Connor, R.; Smith, D.; Fonseka, G.; Al Mutery, A.; Rathje, C.; Frodsham, R et al. Novel tools for characterising inter and intra chromosomal rearrangements in avian microchromosomes. Chromosome Res, 2014; 22:85–97.

13. Sasaki, M. et al. A Feather Pulp Culture Technique for Avian Chromosomes, with Notes on the Chromosomes of the Peafowl and the Ostrich. Experientia, 1968; 24(12):1292-1293.

14. Haring, E.; Kvaloy, K.; Gjershaug, J.; Rov, N. & Gamauf, A. Convergent evolution and paraphyly of the hawk-eagles of the genus *Spizaetus* (Aves, Accipitridae) – phylogenetic analyses based on mitochondrial markers. J Zool Syst Evol Res, 2007; 45(4): 353–365.

15. Romanov, M. N.; Farré, M.; Lithgow, P. E.; Fowler, K. E.; Skinner, B. M.; O'connor, R.; Fonseka, G.; Backström, N.; Matsuda, Y.; Nishida, C et al. Reconstruction of gross avian genome structure, organization and evolution suggests that the chicken lineage most closely resembles the dinosaur avian ancestor. BMC Genomics, 2014; 15:1060.

16. Zhang, G.; Li, C.; Li, Q.; Li, B.; Larkin, D. M.; Lee, C.; Storz, J. F. et al. Comparative genomics reveals insights into avian genome evolution and adaptation. Science, 2014; 346: 1311-1319.

Figure Legends

Figure 1- Metaphase (A) and karyotype (B) of *Spizaetus tyrannus* with diploid number 2n = 68, obtained with Giemsa conventional staining. The red arrows in (A) indicate the sex chromosomes.

Figure 2- Representative results of FISH experiments using *Gallus gallus* chromosome-specific probes corresponding to pairs GGA1 to GGA5 in *Spizaetus tyrannus* karyotype. Red and green signals represent probes labelled with Cy3 or FITC, respectively.

Figure 3- Representative results of hybridizations with some *Gallus gallus* BACs probes in the karyotype of *Spizaetus tyrannus*. (A1 and A1.1) Chicken BAC17 was the only one to hybridize on different chromosomes. Red signals represent probes labeled with Cy3, corresponding to the proximal region (p); Green signals represent probes labeled with FTIC, corresponding to the distal region (d). Arrows indicate the signals.

Figure 4- Idiogram representing the homology between the *Spizaetus tyrannus* chromosomes and the macrochromosome chromosome-specific probes and microchromosomes BAC clones from *Gallus gallus*. Empty boxes mean no signal detected in those chromosomes with the set of probes used. BACs corresponding to pairs 20 and 22 (*) did not produced any detectable signals.

Figure 1

Click here to access/download;Figure;figure01.tif

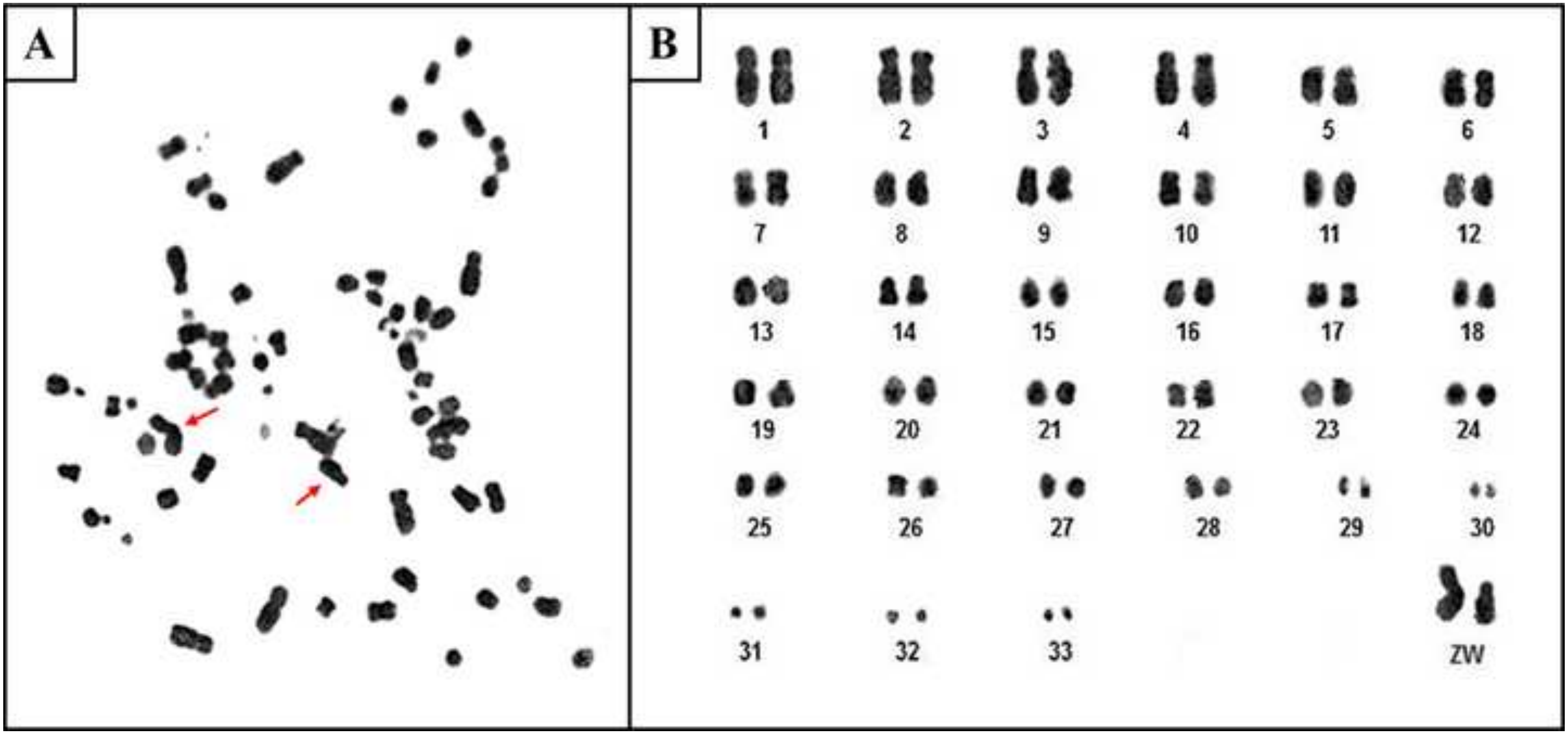

Figure 2

Click here to access/download;Figure;figure02.tif

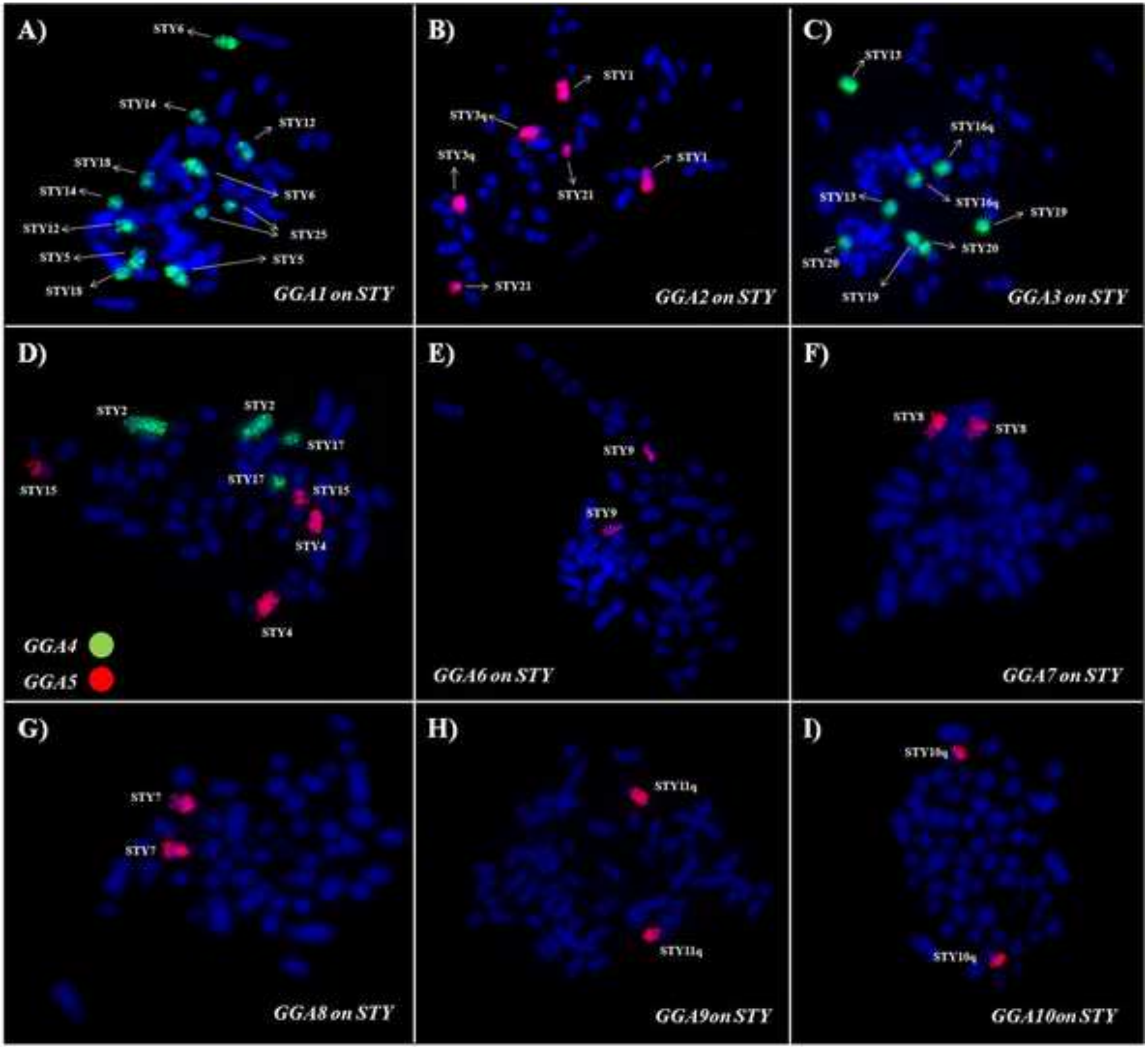

Figure 3

Click here to access/download;Figure;figure03.tif

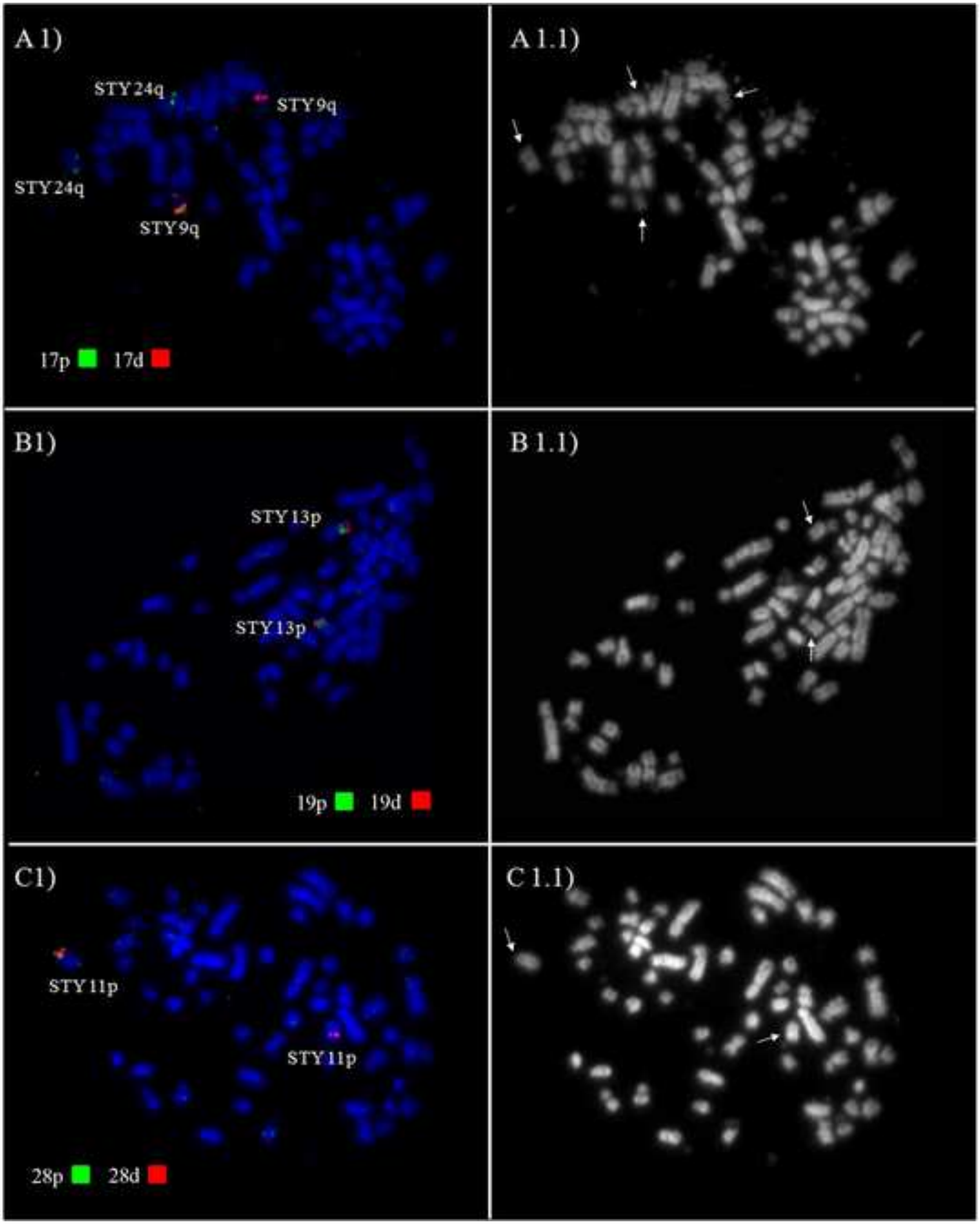

Figure 4

Click here to access/download;Figure;figure04.tif

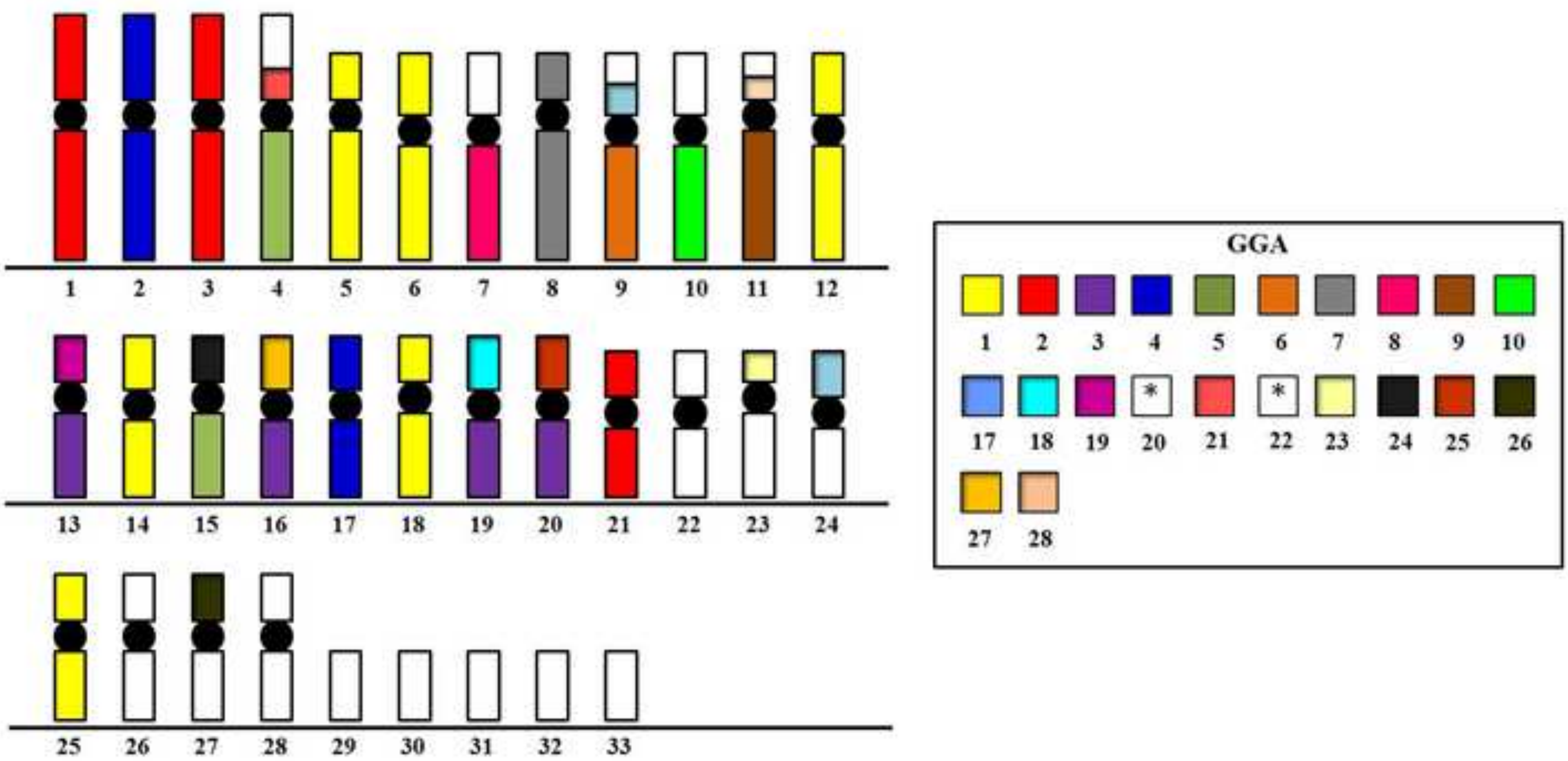

Supplement: Attachment — Submitted filename: PONE-D-21-20639_reviewer.pdf [file pone.0259905.s001.pdf]
