# Supplementary material for: Comparative chromosome painting in Spizaetus tyrannus and Gallus gallus with the use of macro- and microchromosome probes
Source: PLoS One. 2021 Nov 18;16(11):e0259905. doi: 10.1371/journal.pone.0259905 (PMC8601422; doi:10.1371/journal.pone.0259905)
Supplement: Attachment — Submitted filename: Carvalho et al-Response to the reviewers-Plos One.pdf [file pone.0259905.s002.pdf]

UNIVERSIDADE FEDERAL DO PARÁ
INSTITUTO DE CIÊNCIAS EXATAS E NATURAIS
FACULDADE DE CIÊNCIAS NATURAIS

RESPONSE TO REVIEWERS

Belém, September, 17, 2021.

Dear Reviewers,

We are thankful for the constructive reviews that we received; they certainly helped us

to improve the manuscript. Please find below our responses to each of your comments.

Please find below our responses to each of your comments.

Reviewer #1:

1- The work from Carvalho et al brings and interesting analysis in the in the Black-Hawk-Eagle (*Spizaetus tyrannus*) using BAC and WCP experiments, both derived from *Gallus gallus*. The results are very well represented in high-quality figures and the and brings new pieces of information for the big puzzle that avian karyotype evolution represents. Below I indicate some points to be adjusted and put special attention to one suggestion for discussion: The work would profit too much if the analyses were done under a phylogenetic context. An updated phylogeny with the special focus on the phylogenetic position and relation between Chicken and *Spizaetus* would bring more light to the general results found.

A- From the cytotaxonomic point of view, *Gallus gallus* has a basal karyotype, very similar to the avian putative ancestor. As this species is an important biological model, and because it also retained a plesiomorphic karyotype, GGA probes are used as a standard for chromosomal studies in birds. However, except for the use

of other probes, which could reveal intrachromosomal rearrangements, a phylogenetic analysis of GGA and birds of prey is not very informative, except as outgroup

Abstract

2- I missed some introduction about BIRDs cytogenetic in the beginning of Abstract. The authors go too direct about Accipitriformes and most readers do not have such phylogenetic information to which group we are dealing with.

A- We agree, and added an introduction to the abstract "*Although most birds show karyotypes with diploid number around 2n=80, with few macrochromosomes and many microchromosomes pairs, some groups, such as the Accipitriformes, are characterized by a large karyotypic reorganization, which resulted in complements with low diploid numbers, and a smaller number of microchromosomal pairs when compared to other birds*"

3- Why are the authors aiming to investigate homologies with chicken?

A- Because this species is an important biological model, and because it also retained a plesiomorphic karyotype, GGA probes are used as a standard for chromosomal studies in birds. We added a small explanation in the introduction "*In general, studies focusing on chromosome evolution in birds are based in comparative chromosome painting using chicken whole chromosome probes (Gallus gallus – GGA, 2n=78), due to the similarity of the karyotype of this species with the PAK [5].*"

4- Abstract also brings some previous data on NNI that were not originated from this work. I suggest removing.

A- We removed it

5- The terms STY and NNI are used without any previous information/explanation about, causing misunderstandings.

A- We corrected it

6- A final conclusion is missing in the abstract.

A- We added a conclusion in the abstract: "*Comparison with hybridization pattern of the Japanese-Mountain-Eagle, the only Aquilinae analyzed by comparative chromosome painting previously, did not reveal any synapomorphy that could represent a chromosome signature to this subfamily. Therefore, conclusions about karyotype evolution in Aquilinae require additional painting studies*".

Introduction

7- Page3 line3: Present an unusual (delete with)

A- We deleted it.

8- Page 3 line 12: The accurate identification of the chromosomal pairs involved.… if we aim to identify synapomophies….

A- We corrected it

9- Introduction miss a clear motivation for the study.

A- We improved it: "*Among them, the subfamily Aquilinae includes medium and large species, distributed globally, usually known as booted eagle. Usually, ten genera are found within Aquilinae. Cytogenetically, the only information concerning Aquilinae is the definition of the diploid number of six species (four genera), ranging from 2n=66 to 82 [19]".*

10- Why using GGA probes?

A- Because this species is an important biological model, and because it also retained a plesiomorphic karyotype, GGA probes are used as a standard for chromosomal studies in birds. We added a small explanation in the introduction.

11- I missed some info (Figure would be better) about the phylogenetic position and relation between Chicken and *Spizaetus*.

A- The authors agree that for this study are not necessary introduce some information about the phylogenetic position between *Gallus gallus* and *S. tyrannus*, however short information of phylogenetic relationship of the *S.tyrannus* within the Aquilinae Subfamily were added in the introduction aiming improve the reading of this paper.

12- Moreover, inform here as well thar STY corresponds to *Spizaetus tyrannus* on its first mention.

A- This was corrected.

Results

13- Subtitle Karyotypes before the first paragraph.

A- We added it.

Figures

14- Figure 3: I did not understand why using the metaphases present in right column? The signals and chromosomal morphology are clearly visible in the FISH images. Scale bars are missing in all figures.

A- We removed the right column and added the scale bars in all figures.

Reviewer #2:

In this study, the authors used comparative chromosome painting to reveal homology of chromosomal segments between *Spizaetus tyrannus* (the Black-Hawk-Eagle) and *Gallus gallus*. The study is conceptually and methodically motivated. The karyotype of *S. tyrannus* was previously studied only using conventional chromosome staining technique that showed 2n=68 (32m/sm+8st+18a+8m+ZW), and this happened for the first time that the karyotype of this bird of prey was studied by comparative chromosome painting. As a result, the study provides a novel insight into the cytogenetics of the Black-Hawk-Eagle. Both whole chromosome-specific *G. gallus* probes of the 1st-10th pairs and chromosome-specific *G. gallus* BAC probes from 11 pairs of microchromosomes were used. The study evidenced 29 evolutionary fission-fusion events that happened in the evolution of *S. tyrannus* and identified the particular chromosome pairs in the referenced *G. gallus*

karyotype, which have undergone restructuring or have remained unchanged. Another sufficient result concerns the comparison between *S. tyrannus* and *Nisaetus nipalensis orientalis*, which is its only close relative studied so far by comparative chromosome painting. The comparison showed both similarities and differences between the species. The MS is well illustrated by tables and pictures of good quality. In general, the work is interesting and deserves publication in the journal. However, there are some shortcomings in the work. Main disadvantages are (1) almost complete absence of basic data on the karyotypes of the discussed species, which makes it difficult to adequately assess the results obtained, and (2) ignorance of the taxonomic component.

A- We try to improve it.

All other comments that I have are mainly suggestions for improving the article (unfortunately, there is no line numbering in the MS, which makes it difficult for the reviewer to work).

1-Running title. It is too long, should be shorter, e.g., “Comparative Chromosome Painting in *Spizaetus tyrannus* and *Gallus gallus*“.

A- W corrected it.

2. Abstract. Please, decipher GGA, should be *Gallus gallus* (GGA), as done above for *Spizaetus tyrannus* (STY)

A- We corrected it.

Introduction.

3- Page 3. Paragraph 1. Please, provide a putative avian ancestral karyotype, with 2n and the number of microchromosomes.

A- We provided this information in the introduction of the paper.

4- Page 3. Paragraph 1. Here (or further, in Discussion), provide the *G. gallus* karyotype to make further reasoning clearer.

A- We did it.

5- Page 3. Paragraph 3. Unify way of citing – in other places you use numbering.

A- We corrected it.

6- Page 4. Paragraph 4. Please, specify the species studied, *Spizaetus tyrannus*

A- We corrected it.

Discussion.

7- Page 6, Paragraph 1. Please, expand the statement “We report slight differences in chromosome morphology….” by clarifying what Tagliarini et al. (2007) have reported. Describe the differences, give for comparison one and the other karyotypes (otherwise, you only have a declaration).

A- In order to clarify the difference in chromosome morphology described between this study and the Tagliarini et al., (2007), we made a comparative table that was introduce in the results.

8- Page 7. Paragraph 3. The Japanese mountain hawk-eagle *Nisaetus nipalensis orientalis* (Temminck & Schlegel, 1844), not the Hodgson's hawk-eagle *Nisaetus nipalensis* Hodgson, 1836.

A- We corrected it.

9- Other remarks can be found in the MS attached.

A- We followed all the instructions of attached.

Reviewer #3:

This study provides a cytogenetic mapping of the Black-Hawk-Eagle (*Spizaetus tyrannus*) using whole-chromosome paints and BAC probes of *Gallus gallus*. Using this

cytogenetic mapping, the authors investigate the chromosome homologies between *Spizaetus tyrannus* and *Gallus gallus*. Overall the manuscript presents an advance in cytogenetic of Accipitriformes. The manuscript is written in understandable English, but some procedures require more details. Please see below my comments that could help to further enhance the quality of the study.

2) Abstract: “chicken (*Gallus gallus* – GGA)”.

A- We corrected it.

3) Introduction and discussion: The manuscript is written in a manner suitable for a more specialized journal such as Cytogenetic and Genome Research or Comparative Cytogenetics, but not ideally for a general journal such as PlosOne. The introduction and discussion are rather limited to the focus of the analyses. This is a shame as I think the authors did a good job to gather nice cytogenetic results. Thus, the authors should consider redrafting these sections on a broad context.

The introduction could be more informative about the study as a whole. For example, the authors can give information about the contribution of this kind of work to karyotypic evolution and chromosomal organization of Accipitriformes. In this line, the last paragraph of introduction can be better written showing the importance of this study, and not merely comment “This study presents the cytogenetic mapping of a species…”. Moreover, other intriguing topic is about the origin of microchromosomes. And maybe the authors could comment a little bit about this topic in the introduction and also discussion. Below is a reference related to this topic.

- Waters, P. D., Patel, H. R., Ruíz-Herrera, A., Álvarez-González, L., Lister, N. C., Simakov, O., ... & Graves, J. A. M. (2021). Microchromosomes are building blocks of bird, reptile and mammal chromosomes. bioRxiv.

A- The introduction was rewritten and several new information were added to improve the understanding of the manuscript. Also, some information from the reference recommended by the reviewer were considerate in this topic.

4) Results, paragraph 1: The authors commented that they detected “four pairs of microchromosomes” but they indicated five (29, 30, 31, 32, and 33). Considering the Figure 1B the pair 29 apparently is not a microchromosome.

A- We reviewed the Karyotype of *S.tyrannus* and considered only four pairs of microchromosomes (Pairs: 30, 31, 32 and 33). Also, we corrected it throughout of the text.

5) Results, paragraph 2: “The most extreme examples are the fission of GGA1 into six pairs in STY, and GGA3 into three distinct pairs”. It seems that for GGA3 are four pairs (13, 16, 19, and 20) (Figure 4), right?

A- The reviewer is right. The GGA3 probe correspond to four pairs (13,16,19 and 20).
We changed it.

6) Discussion, paragraph 3: “…karyotype of STY when compared to *Gallus*”; “…karyotype of STY when compared to *Gallus gallus*”.

A- We corrected it.

7) Discussion, paragraph 4: “…microchromosomes (STY4, 7 and 9; NNI2, 4 and 9), none of them”; “microchromosomes (STY: pairs 4, 7 and 9; NNI: pairs 2, 4 and 9), none of them”.
“…with microchromosomes (GGA: pairs 18, 19, 24 and 25)”.

A- We corrected it.

8) Discussion, paragraph 5: “These results show that they are morphologically similar species that until the last decade were part of the same genus [14].”. I think the authors results did not show an association of chromosomal data with morphological similarity and this should be corrected.

A- We removed it.

9) Conclusion: "…of *S.tyrannus* should be…"; "…of *S. tyrannus* should be…".

A- We corrected it.

10) Methods, paragraph 1: What the name and country of the Zoos?

A- The information was added in the Methods.

11) Methods, paragraph 2: "…and labelled directly by FTIC"; "and labelled directly by fluorescein isothiocyanate (FITC)".

A- We changed it.

12) Methods: the results of this study are based on FISH with GGA probes. However, FISH procedure is not sufficiently described. I would appreciate if at least main/important steps of the FISH procedure are described. This would also avoid any doubt about the accuracy of the results.

A- We provided the main steps of the FISH procedure.

13) Figure 3: "FITC".

A- We corrected it.

14) Please, give a scale bar information for all figures. It is very important, specially considering the presence of macrochromosomes and microchromosomes.

A- We added the scale bar in all figures.
