# Supplementary material for: Comparative chromosome painting in Spizaetus tyrannus and Gallus gallus with the use of macro- and microchromosome probes
Source: PLoS One. 2021 Nov 18;16(11):e0259905. doi: 10.1371/journal.pone.0259905 (PMC8601422; doi:10.1371/journal.pone.0259905)
Supplement: Attachment — Submitted filename: PONE-D-21-20639_R1_rev.pdf [file pone.0259905.s003.pdf]

PLOS ONE

Comparative Chromosome Painting in the Black-Hawk-Eagle (Spizaetus tyrannus) and Gallus gallus with the use of Macro and Microchromosome Probes.

--Manuscript Draft--

**Manuscript Number:**	PONE-D-21-20639R1
**Article Type:**	Research Article
**Full Title:**	Comparative Chromosome Painting in the Black-Hawk-Eagle (Spizaetus tyrannus) and Gallus gallus with the use of Macro and Microchromosome Probes.
**Short Title:**	Comparative Chromosome Painting in the Black-Hawk-Eagle (Spizaetus tyrannus) and Gallus gallus
**Corresponding Author:**	Edivaldo Herculano Corrêa de Oliveira<br>Universidade Federal do Pará<br>Belém, Para BRAZIL
**Keywords:**	Aquilinae; Spizaetus; chromosome painting; BACs; Microchromosomes
**Abstract:**	Although most birds show karyotypes with diploid number around 2n=80, with few macrochromosomes and many microchromosomes pairs, some groups, such as the Accipitriformes, are characterized by a large karyotypic reorganization, which resulted in complements with low diploid numbers, and a smaller number of microchromosomal pairs when compared to other birds. Among Accipitriformes, the Accipitridae family is the most diverse and includes the subfamily Aquilinae, composed of medium to large sized species. The Black-Hawk-Eagle (Spizaetus tyrannus - STY), found in South America, is a member of this subfamily. Available chromosome data for this species includes only conventional staining. Hence, in order to provide additional information on karyotype evolution process within this group, we performed comparative chromosome painting between S. tyrannus and Gallus gallus (GGA). Our results revealed that at least 29 fission-fusion events were observed in the STY karyotype, based on homology with GGA. Fissions occurred mainly in syntenic groups homologous to GGA1-GGA5. On the other hand, the majority of the microchromosomes were found fused to other chromosomal elements in STY, indicating these rearrangements had an important role in the reduction of the diploid number to 2n=68. Comparison with hybridization pattern of the Japanese-Mountain-Eagle, the only Aquilinae analyzed by comparative chromosome painting previously, did not reveal any synapomorphy that could represent a chromosome signature to this subfamily. Therefore, conclusions about karyotype evolution in Aquilinae require additional painting studies.
**Order of Authors:**	Carlos A Carvalho
	Ivanete O Furo
	Patricia C.M. O´Brien
	Jorge Pereira
	Rebeca E O´Connor
	Darren Griffin
	Malcolm Ferguson-Smith
	Edivaldo Herculano Corrêa de Oliveira
**Response to Reviewers:**	Dear Editor and reviewer<br>We are thankful for the constructive reviews that we received; they certainly helped us to improve the manuscript. Please find below our responses to each of your comments.<br>Sincerely,<br><br>Reviewer #1:<br>1- The work from Carvalho et al brings and interesting analysis in the in the Black-Hawk-Eagle (Spizaetus tyrannus) using BAC and WCP experiments, both derived from Gallus gallus. The results are very well represented in high-quality figures and the and

Powered by Editorial Manager® and ProduXion Manager® from Aries Systems Corporation

brings new pieces of information for the big puzzle that avian karyotype evolution represents. Below I indicate some points to be adjusted and put special attention to one suggestion for discussion: The work would profit too much if the analyses were done under a phylogenetic context. An updated phylogeny with the special focus on the phylogenetic position and relation between Chicken and Spizaetus would bring more light to the general results found.
A-From the cytotaxonomic point of view, Gallus gallus has a basal karyotype, very similar to the avian putative ancestor. As this species is an important biological model, and because it also retained a plesiomorphic karyotype, GGA probes are used as a standard for chromosomal studies in birds. However, except for the use of other probes, which could reveal intrachromosomal rearrangements, a phylogenetic analysis of GGA and birds of prey is not very informative, except as outgroup

Abstract
2- I missed some introduction about BIRDs cytogenetic in the beginning of Abstract. The authors go too direct about Accipitriformes and most readers do not have such phylogenetic information to which group we are dealing with.
A-We agree, and added an introduction to the abstract "Although most birds show karyotypes with diploid number around 2n=80, with few macrochromosomes and many microchromosomes pairs, some groups, such as the Accipitriformes, are characterized by a large karyotypic reorganization, which resulted in complements with low diploid numbers, and a smaller number of microchromosomal pairs when compared to other birds"

3- Why are the authors aiming to investigate homologies with chicken?
A-Because this species is an important biological model, and because it also retained a plesiomorphic karyotype, GGA probes are used as a standard for chromosomal studies in birds. We added a small explanation in the introduction "In general, studies focusing on chromosome evolution in birds are based in comparative chromosome painting using chicken whole chromosome probes (Gallus gallus – GGA, 2n=78), due to the similarity of the karyotype of this species with the PAK [5]."
4- Abstract also brings some previous data on NNI that were not originated from this work. I suggest removing.
A-We removed it
5- The terms STY and NNI are used without any previous information/explanation about, causing misunderstandings.
A-We corrected it

6- A final conclusion is missing in the abstract.
A-We added a conclusion in the abstract: "Comparison with hybridization pattern of the Japanese-Mountain-Eagle, the only Aquilinae analyzed by comparative chromosome painting previously, did not reveal any synapomorphy that could represent a chromosome signature to this subfamily. Therefore, conclusions about karyotype evolution in Aquilinae require additional painting studies".

Introduction
7- Page3 line3: Present an unusual (delete with)
A-We deleted it.

8- Page 3 line 12: The accurate identification of the chromosomal pairs involved…. if we aim to identify synapomophies….
A-We corrected it

9- Introduction miss a clear motivation for the study.
A-We improved it: "Among them, the subfamily Aquilinae includes medium and large species, distributed globally, usually known as booted eagle. Usually, ten genera are found within Aquilinae. Cytogenetically, the only information concerning Aquilinae is the definition of the diploid number of six species (four genera), ranging from 2n=66 to 82 [19]".

10- Why using GGA probes?
A-Because this species is an important biological model, and because it also retained a plesiomorphic karyotype, GGA probes are used as a standard for chromosomal studies in birds. We added a small explanation in the introduction.

11- I missed some info (Figure would be better) about the phylogenetic position and relation between Chicken and Spizaetus.
A-The authors agree that for this study are not necessary introduce some information about the phylogenetic position between Gallus gallus and S. tyrannus, however short information of phylogenetic relationship of the S.tyrannus within the Aquilinae Subfamily were added in the introduction aiming improve the reading of this paper.
12- Moreover, inform here as well thar STY corresponds to Spizaetus tyrannus on its first mention.
A-This was corrected.
Results
13- Subtitle Karyotypes before the first paragraph.
A-We added it.
Figures
14- Figure 3: I did not understand why using the metaphases present in right column? The signals and chromosomal morphology are clearly visible in the FISH images. Scale bars are missing in all figures.
A- We removed the right column and added the scale bars in all figures.

Reviewer #2:
In this study, the authors used comparative chromosome painting to reveal homology of chromosomal segments between Spizaetus tyrannus (the Black-Hawk-Eagle) and Gallus gallus. The study is conceptually and methodically motivated. The karyotype of S. tyrannus was previously studied only using conventional chromosome staining technique that showed 2n=68 (32m/sm+8st+18a+8m+ZW), and this happened for the first time that the karyotype of this bird of prey was studied by comparative chromosome painting. As a result, the study provides a novel insight into the cytogenetics of the Black-Hawk-Eagle. Both whole chromosome-specific G. gallus probes of the 1st-10th pairs and chromosome-specific G. gallus BAC probes from 11 pairs of microchromosomes were used. The study evidenced 29 evolutionary fission-fusion events that happened in the evolution of S. tyrannus and identified the particular chromosome pairs in the referenced G. gallus karyotype, which have undergone restructuring or have remained unchanged. Another sufficient result concerns the comparison between S. tyrannus and Nisaetus nipalensis orientalis, which is its only close relative studied so far by comparative chromosome painting. The comparison showed both similarities and differences between the species. The MS is well illustrated by tables and pictures of good quality.
In general, the work is interesting and deserves publication in the journal.
However, there are some shortcomings in the work. Main disadvantages are (1) almost complete absence of basic data on the karyotypes of the discussed species, which makes it difficult to adequately assess the results obtained, and (2) ignorance of the taxonomic component.
A- We try to improve it.

All other comments that I have are mainly suggestions for improving the article (unfortunately, there is no line numbering in the MS, which makes it difficult for the reviewer to work).

1-Running title. It is too long, should be shorter, e.g., “Comparative Chromosome Painting in Spizaetus tyrannus and Gallus gallus“.
A-W corrected it.
2. Abstract. Please, decipher GGA, should be Gallus gallus (GGA), as done above for Spizaetus tyrannus (STY)
A- We corrected it.

Introduction.

3- Page 3. Paragraph 1. Please, provide a putative avian ancestral karyotype, with 2n and the number of microchromosomes.
A-We provided this information in the introduction of the paper.
4- Page 3. Paragraph 1. Here (or further, in Discussion), provide the G. gallus karyotype to make further reasoning clearer.
A-We did it.

5- Page 3. Paragraph 3. Unify way of citing – in other places you use numbering.

Powered by Editorial Manager® and ProduXion Manager® from Aries Systems Corporation

A-We corrected it.

6- Page 4. Paragraph 4. Please, specify the species studied, Spizaetus tyrannus
A-We corrected it.

Discussion.

7- Page 6, Paragraph 1. Please, expand the statement “We report slight differences in chromosome morphology….” by clarifying what Tagliarini et al. (2007) have reported. Describe the differences, give for comparison one and the other karyotypes (otherwise, you only have a declaration).

A-In order to clarify the difference in chromosome morphology described between this study and the Tagliarini et al., (2007), we made a comparative table that was introduce in the results.
8- Page 7. Paragraph 3. The Japanese mountain hawk-eagle Nisaetus nipalensis orientalis (Temminck & Schlegel, 1844), not the Hodgson's hawk-eagle Nisaetus nipalensis Hodgson, 1836.

A- We corrected it.
9- Other remarks can be found in the MS attached.
A-We followed all the instructions of attached.

Reviewer #3:
This study provides a cytogenetic mapping of the Black-Hawk-Eagle (Spizaetus tyrannus) using whole-chromosome paints and BAC probes of Gallus gallus. Using this cytogenetic mapping, the authors investigate the chromosome homologies between Spizaetus tyrannus and Gallus gallus. Overall the manuscript presents an advance in cytogenetic of Accipitriformes. The manuscript is written in understandable English, but some procedures require more details. Please see below my
comments that could help to further enhance the quality of the study.
2) Abstract: “chicken (Gallus gallus – GGA)”.
A- We corrected it.

3) Introduction and discussion: The manuscript is written in a manner suitable for a more specialized journal such as Cytogenetic and Genome Research or Comparative Cytogenetics, but not ideally for a general journal such as PlosOne. The introduction and discussion are rather limited to the focus of the analyses. This is a shame as I think the authors did a good job to gather nice cytogenetic results. Thus, the authors should consider redrafting these sections on a broad context.

The introduction could be more informative about the study as a whole. For example, the authors can give information about the contribution of this kind of work to karyotypic evolution and chromosomal organization of Accipitriformes. In this line, the last paragraph of introduction can be better written showing the importance of this study, and not merely comment “This study presents the cytogenetic mapping of a species…”. Moreover, other intriguing topic is about the origin of microchromosomes. And maybe the authors could comment a little bit about this topic in the introduction and also discussion. Below is a reference related to this topic.

- Waters, P. D., Patel, H. R., Ruíz-Herrera, A., Álvarez-González, L., Lister, N. C., Simakov, O., ... & Graves, J. A. M. (2021). Microchromosomes are building blocks of bird, reptile and mammal chromosomes. bioRxiv.

A-The introduction was rewritten and several new information were added to improve the understanding of the manuscript. Also, some information from the reference recommended by the reviewer were considerate in this topic.

4) Results, paragraph 1: The authors commented that they detected “four pairs of microchromosomes” but they indicated five (29, 30, 31, 32, and 33). Considering the Figure 1B the pair 29 apparently is not a microchromosome.

A-We reviewed the Karyotype of S.tyrannus and considered only four pairs of microchromosomes (Pairs: 30, 31, 32 and 33). Also, we corrected it throughout of the

	text.<br><br>5) Results, paragraph 2: “The most extreme examples are the fission of GGA1 into six pairs in STY, and GGA3 into three distinct pairs”. It seems that for GGA3 are four pairs (13, 16, 19, and 20) (Figure 4), right?<br><br>A-The reviewer is right. The GGA3 probe correspond to four pairs (13,16,19 and 20). We changed it.<br><br>6) Discussion, paragraph 3: “…karyotype of STY when compared to Gallus”; “…karyotype of STY when compared to Gallus gallus”.<br><br>A-We corrected it.<br>7) Discussion, paragraph 4: “…microchromosomes (STY4, 7 and 9; NNI2, 4 and 9), none of them”; “microchromosomes (STY: pairs 4, 7 and 9; NNI: pairs 2, 4 and 9), none of them”.<br>“…with microchromosomes (GGA: pairs 18, 19, 24 and 25)”.<br><br>A-We corrected it.<br><br>8) Discussion, paragraph 5: “These results show that they are morphologically similar species that until the last decade were part of the same genus [14].”. I think the authors results did not show an association of chromosomal data with morphological similarity and this should be corrected.<br><br>A-We removed it.<br><br>9) Conclusion: “…of S.tyrannus should be…”; “…of S. tyrannus should be…”.<br><br>A-We corrected it.<br><br>10) Methods, paragraph 1: What the name and country of the Zoos?<br><br>A-The information was added in the Methods.<br><br>11) Methods, paragraph 2: “…and labelled directly by FTIC”; “and labelled directly by fluorescein isothiocyanate (FITC)”.<br><br>A-We changed it.<br><br>12) Methods: the results of this study are based on FISH with GGA probes. However, FISH procedure is not sufficiently described. I would appreciate if at least main/important steps of the FISH procedure are described. This would also avoid any doubt about the accuracy of the results.<br><br>A- We provided the main steps of the FISH procedure.<br>13) Figure 3: “FITC”.<br>A- We corrected it.<br><br>14) Please, give a scale bar information for all figures. It is very important, specially considering the presence of macrochromosomes and microchromosomes.<br>A-We added the scale bar in all figures.
Additional Information:	
Question	Response
**Financial Disclosure**<br><br>Enter a financial disclosure statement that describes the sources of funding for the work included in this submission. Review the submission guidelines for detailed requirements. View published research	This research was partially funded by a grant to EHCO from CNPq (307382/2019-2) and to MAFS from the Wellcome Trust in support of the Cambridge Resource Centre for Comparative Genomics and by the Biotechnology and Biological Sciences Research Council (BB/K008161/1) to the University of Kent.

articles from *PLOS ONE* for specific examples.<br><br>This statement is required for submission and **will appear in the published article** if the submission is accepted. Please make sure it is accurate.<br><br>**Unfunded studies**<br>Enter: *The author(s) received no specific funding for this work.*<br><br>**Funded studies**<br>Enter a statement with the following details:<br>• Initials of the authors who received each award<br>• Grant numbers awarded to each author<br>• The full name of each funder<br>• URL of each funder website<br>• Did the sponsors or funders play any role in the study design, data collection and analysis, decision to publish, or preparation of the manuscript?<br>• **NO** - Include this sentence at the end of your statement: *The funders had no role in study design, data collection and analysis, decision to publish, or preparation of the manuscript.*<br>• **YES** - Specify the role(s) played.<br><br>* typeset	
**Competing Interests**<br><br>Use the instructions below to enter a competing interest statement for this submission. On behalf of all authors, disclose any competing interests that could be perceived to bias this work—acknowledging all financial support and any other relevant financial or non-financial competing interests.<br><br>This statement is required for submission and **will appear in the published article** if the submission is accepted. Please make sure it is accurate and that any funding sources listed in your Funding Information later in the submission form are also declared in your Financial Disclosure statement.	The authors have declared that no competing interests exist.

Powered by Editorial Manager® and ProduXion Manager® from Aries Systems Corporation

View published research articles from *PLOS ONE* for specific examples.<br><br>**NO authors have competing interests**<br><br>Enter: *The authors have declared that no competing interests exist.*<br><br>**Authors with competing interests**<br><br>Enter competing interest details beginning with this statement:<br><br>*I have read the journal's policy and the authors of this manuscript have the following competing interests: [insert competing interests here]*<br><br>* typeset	
**Ethics Statement**<br><br>Enter an ethics statement for this submission. This statement is required if the study involved:<br><br>• Human participants<br>• Human specimens or tissue<br>• Vertebrate animals or cephalopods<br>• Vertebrate embryos or tissues<br>• Field research<br><br>Write "N/A" if the submission does not require an ethics statement.<br><br>General guidance is provided below. Consult the submission guidelines for detailed instructions. **Make sure that all information entered here is included in the Methods section of the manuscript.**	The experiments were carried out according to the ethical protocols approved by an ethics committee (CEUA—Federal University of Pará) under no. 170/2013 and SISBIO 68443-1.

Powered by Editorial Manager® and ProduXion Manager® from Aries Systems Corporation

**Format for specific study types**<br><br>**Human Subject Research (involving human participants and/or tissue)**<br>• Give the name of the institutional review board or ethics committee that approved the study<br>• Include the approval number and/or a statement indicating approval of this research<br>• Indicate the form of consent obtained (written/oral) or the reason that consent was not obtained (e.g. the data were analyzed anonymously)<br><br>**Animal Research (involving vertebrate animals, embryos or tissues)**<br>• Provide the name of the Institutional Animal Care and Use Committee (IACUC) or other relevant ethics board that reviewed the study protocol, and indicate whether they approved this research or granted a formal waiver of ethical approval<br>• Include an approval number if one was obtained<br>• If the study involved *non-human primates*, add *additional details* about animal welfare and steps taken to ameliorate suffering<br>• If anesthesia, euthanasia, or any kind of animal sacrifice is part of the study, include briefly which substances and/or methods were applied<br><br>**Field Research**<br><br>Include the following details if this study involves the collection of plant, animal, or other materials from a natural setting:<br>• Field permit number<br>• Name of the institution or relevant body that granted permission	
**Data Availability**<br><br>Authors are required to make all data underlying the findings described fully available, without restriction, and from the time of publication. PLOS allows rare exceptions to address legal and ethical concerns. See the PLOS Data Policy and FAQ for detailed information.	Yes - all data are fully available without restriction

A Data Availability Statement describing where the data can be found is required at submission. Your answers to this question constitute the Data Availability Statement and **will be published in the article**, if accepted.<br><br>**Important:** Stating 'data available on request from the author' is not sufficient. If your data are only available upon request, select 'No' for the first question and explain your exceptional situation in the text box.<br><br>Do the authors confirm that all data underlying the findings described in their manuscript are fully available without restriction?	
**Describe where the data may be found in full sentences. If you are copying our sample text, replace any instances of XXX with the appropriate details.**<br><br>• If the data are **held or will be held in a public repository**, include URLs, accession numbers or DOIs. If this information will only be available after acceptance, indicate this by ticking the box below. For example: *All XXX files are available from the XXX database (accession number(s) XXX, XXX.).*<br>• If the data are all contained **within the manuscript and/or Supporting Information files**, enter the following: *All relevant data are within the manuscript and its Supporting Information files.*<br>• If neither of these applies but you are able to provide **details of access elsewhere**, with or without limitations, please do so. For example:<br><br>*Data cannot be shared publicly because of [XXX]. Data are available from the XXX Institutional Data Access / Ethics Committee (contact via XXX) for researchers who meet the criteria for access to confidential data.*<br><br>*The data underlying the results presented in the study are available from (include the name of the third party*	All relevant data are within the manuscript and its Supporting Information files. All conditions for experiments are presented in the text.

and contact information or URL).<br>• This text is appropriate if the data are owned by a third party and authors do not have permission to share the data.<br><br>* typeset	
Additional data availability information:	

Manuscript

Click here to access/download;Manuscript;Carvalho et al. Manuscript-revised version2.docx

Comparative Chromosome Painting in the Black-Hawk-Eagle (*Spizaetus tyrannus*) and *Gallus gallus* with the use of Macro and Microchromosome Probes.

Short Title: Comparative Chromosome Painting in *Spizaetus tyrannus* and *Gallus gallus*

Carlos Augusto L Carvalho[1, 2], Ivanete de Oliveira Furo [2, 3], Patricia C. M. O'Brien[4], Jorge Pereira J [5], Rebeca E. O'Connor [6], Darren Griffin[6], Malcolm A. Ferguson-Smith MA[4], Edivaldo Herculano C. de Oliveira[3,7] *

1 Programa de Pós-Graduação em Genética e Biologia Molecular, Universidade Federal do Pará, Belém, Pará 66075-110, Brazil

2 Laboratório de Citogenômica e Mutagênese Ambiental, SAMAM, Instituto Evandro Chagas, Ananindeua, Pará 67030-000, Brazil

3 Universidade Federal Rural da Amazônia (UFRA) Laboratório de Reprodução Animal (LABRAC), Parauapebas, Pará, 68515-000, Brazil

4 Cambridge Resource Centre for Comparative Genomics, Cambridge CB3 0ES, UK

5 Animal and Veterinary Research Center, Universidade de Trá-os-Montes e Alto douro, Vila Real, Portugal

6 School of Biosciences, University of Kent, Canterbury CT2 7NJ, UK

7 Faculdade de Ciências Naturais, Instituto de Ciências Exatas e Naturais, Universidade Federal do Pará, Belém, Pará 66075-110, Brazil

Corresponding Author:

***Dr. Edivaldo Herculano C. de Oliveira**

ehco@ufpa.br, edivaldooliveira@iec.gov.br

Abstract

Although most birds show karyotypes with diploid number around 2n=80, with few macrochromosomes and many microchromosomes pairs, some groups, such as the Accipitriformes, are characterized by a large karyotypic reorganization, which resulted in complements with low diploid numbers, and a smaller number of microchromosomal pairs when compared to other birds. Among Accipitriformes, the Accipitridae family is the most diverse and includes the subfamily Aquilinae, composed of medium to large sized species. The Black-Hawk-Eagle (*Spizaetus tyrannus* - STY), found in South America, is a member of this subfamily. Available chromosome data for this species includes only conventional staining. Hence, in order to provide additional information on karyotype evolution process within this group, we performed comparative chromosome painting between *S. tyrannus* and *Gallus gallus* (GGA). Our results revealed that at least 29 fission-fusion events were observed in the STY karyotype, based on homology with GGA. Fissions occurred mainly in syntenic groups homologous to GGA1-GGA5. On the other hand, the majority of the microchromosomes were found fused to other chromosomal elements in STY, indicating these rearrangements had an important role in the reduction of the diploid number to 2n=68. Comparison with hybridization pattern of the Japanese-Mountain-Eagle, the only Aquilinae analyzed by comparative chromosome painting previously, did not reveal any synapomorphy that could represent a chromosome signature to this subfamily. Therefore, conclusions about karyotype evolution in Aquilinae require additional painting studies.

Key Words: Aquilinae, Chromosome painting, BACs, Microchromosomes

Introduction

Usually, bird genome is organized in karyotypes consisting of few macrochromosomes and many tiny microchromosomes [1]. However, there are some exceptions. For instance, excluding the New World vultures (Cathartidae), which show similar karyotypes to the putative avian ancestral karyotype (PAK) with diploid number around 2n [illegible] 80, including 10 pairs of macrochromosomes and 30 pairs of microchromosomes [1], species belonging to the Order Accipitriformes present an interesting chromosomal diversity. They have lower diploid numbers, 2n = 54-68, and a reduction of microchromosomes to between 4 and 8 pairs, due mainly to fusions involving these small elements, occurred during their divergence [2-4].

In general, studies focusing on chromosome evolution in birds are based in comparative chromosome painting using chicken whole chromosome probes (*Gallus gallus* – GGA, 2n=78), due to the similarity of the karyotype of this species with the PAK [5]. The use of this methodology in species of birds of prey has revealed that, despite the lower diploid numbers observed in this group, the large karyotype reorganization in Accipitriformes included multiple fissions in the macrochromosome pairs homologous to GGA1-GGA5. The reduction of the chromosome number would be due to the concomitant occurrence of several fusion events involving microchromosomes [6-11].

Microchromosomes are gene rich elements, and genome comparative analyses have shown their conservation as syntenic groups among distantly related bird groups [12-13]. In fact, rearrangements involving microchromosomes were detected in few orders: Psittaciformes, Cuculiformes, Suliformes, Caprimulgiformes and the Accipitriformes [13-15]. Due to difficulties of the isolation of individual microchromosome pairs by flow cytometry for specific probe production, most data concerning microchromosomes were obtained by the use of pools of microchromosomes,

i.e., chromosome paints that recognize more than one pair. Therefore, improved identification of chromosome pairs involved in rearrangements is a priority if we are to achieve a more definitive analysis and identify synapomorphies based on chromosome characters [16-17].

Currently, the order Accipitriformes is composed of four families, of which Accipitridae is the most diverse, with approximately 230 species distributed in 14 subfamilies [18]. Among them, the subfamily Aquilinae includes medium and large species, distributed globally, usually known as booted eagle. Usually, ten genera are found within Aquilinae. Cytogenetically, the only information concerning Aquilinae is the definition of the diploid number of six species (four genera), ranging from 2n=66 to 82 [19].

The Black-Hawk-Eagle (*Spizaetus tyrannus*-STY) is a representative of this subfamily, found in South and Central Americas, from southern Mexico down to Argentina [18]. Considering that the only chromosomal analysis of *S. tyrannus* to date was based on conventional staining, revealing a karyotype within the Aquilinae standard, with 2n=68 [1], the aim of this study was to present the cytogenetic mapping of *S. tyrannus* by comparative painting. In addition to whole-chromosome paints of *Gallus gallus* (GGA), we used BAC probes from GGA clones that identified 11 individual pairs of microchromosomes. The results were compared to *Nisaetus nipalensis orientalis*- NNI (2n=66) [10], also from the subfamily Aquilinae, in order to identify chromosomal rearrangements related to karyotype evolution in this group.

Results

Karyotype description

The karyotype of *Spizaetus tyrannus* presented a dipl[illegible] number 2n=68, consisting of 21 me[illegible]r submetacentric pairs (pairs 1-4, 6-7, 9-10, 12, 14, 16-17, 19-22, 24-29 and the sex chromosom[illegible]), seven acrocentric (pairs 5, 8, 11, 13, 15, 18 and 23), and four pairs of microchromosomes (pairs 30-33). The Z chromosome is a large metacentric, with size between pairs 3 or 4, while the W chromosome is an average submetacentric, similar in size to pairs 8 or 9 (Figure 1). In table 1, we reported some differences in chromosome morphology of *S. tyrannus* described by Tagliarini et al., [1] (Table 1).

Table 1- Karyotype of *S. tyrannus* described by Tagliarini et a[illegible]nd at this study. (Metacentric: M; Submetacentric: SM; Subtelocentric: ST; Acrocentric: AC)

**Pairs**	**This study**	**Tagliarini et al., 2007**	**Pairs**	**This study**	**Tagliarini et al., 2[illegible]**
**Chr 1.**	SM	SM	Chr 18.	AC	AC
**Chr 2.**	SM	SM	Chr 19.	SM	SM
**Chr 3.**	SM	SM	Chr 20.	SM	SM
**Chr 4.**	SM	SM	Chr 21.	SM	SM
**Chr 5.**	AC	SM	Chr 22.	SM	SM
**Chr 6.**	SM	ST	Chr 23.	AC	AC
**Chr 7.**	SM	SM	Chr 24.	SM	AC
**Chr 8.**	AC	ST	Chr 25.	SM	AC
**Chr 9.**	SM	SM	Chr 26.	SM	AC
**Chr 10.**	SM	ST	Chr 27.	SM	SM
**Chr 11.**	AC	SM	Chr 28.	SM	AC
**Chr 12.**	SM	SM	Chr 29.	SM	SM
**Chr 13.**	AC	SM	Chr 30.	Micro	Micro
**Chr 14.**	SM	AC	Chr 31.	Micro	Micro
**Chr 15.**	AC	AC	Chr 32.	Micro	Micro
**Chr 16.**	SM	ST	Chr 33.	Micro	Micro
**Chr 17.**	SM	ST	Chr ZW.	M and SM	M and SM

Comparative Chromosome painting

G. gallus probes used in the fluorescent *in situ* hybridization (FISH) experiments produced reproducible results. Hybridizations with chromosome-specific probes for the first ten pairs of GGA produced 22 signals, with the first five pairs producing multiple signals, ranging from 2 to 6 signals (Figure 2). For instance, GGA1 probe painted six distinct pairs in the *S. tyrannus* karyotype (pairs 5, 6, 12, 14, 18, and 25), while the probes GGA6-10 pairs showed only one signal each. Table 2 details the distribution of the signals produced by GGA whole specific probes in the karyotype of *S. tyrannus*.

Table 2- Results of hybridizations with *G. gallus* probes showing the homology between GGA probes in the karyotype of *S. tyrannus* (STY).

Probes	STY Chromosomes	Probes	STY Chromosomes
**GGA1**	(5, 6, 12, 14, 18, 25)	**GGA6**	9
**GGA2**	(1, 3q, 21)	**GGA7**	8
**GGA3**	(13, 16q, 19, 20)	**GGA8**	7
**GGA4**	(2, 17)	**GGA9**	11q
**GGA5**	(4, 15q)	**GGA10**	10q

A total of 19 out of 22 *G. gallus* BAC clones produced results. Both BACs from the GGA22 chromosome did not produce any detectable signal, as well as one of the BACs from GGA21. Among the 19 probes that gave good quality results, both proximal (BACp) and distal (BACd) referring to 8 pairs, were found in the same segment in the STY karyotype. However, BACs corresponding to GGA17 hybridized to two different pairs - BAC17p marked STY 9q, while BAC17d marked STY 24q. (Figure 3). All Chicken BACs and their respective homology in the karyotype of *S. tyrannus* are summarized in Table 3.

Table 3- Summary of the results of experiments using GGA BACs on the karyotype of *S. tyrannus* (*=BACs marking the same segment in STY karyotype; p=proximal region; d=distal region).

GGA	BAC ID	STY	GGA	BAC ID	STY
**17p**	CH261-113A7	[illegible]	**23d**	CH261-90K11	23p*
**17d**	CH261-42P16	24q	**24p**	CH261-103F4	15p*
**18p**	CH261-60N6	19p*	**24d**	CH261-65O4	15p*
**18d**	CH261-72B18	19p*	**25p**	CH261-59C21	20p*
**19p**	CH261-10F1	13p*	**25d**	CH261-127K7	20p*
**19d**	CH261-50H12	13p*	**26p**	CH261-186M13	27p*
**21p**	CH261-83I20	No signal	**26d**	CH261-170L23	27p*
**21d**	CH261-122K8	4p	**27p**	CH261-66M16	16p*
**22p**	CH261-40J9	No signal	**27d**	CH261-28L10	16p*
**22d**	CH261-18G17	No signal	**28p**	CH261-64A15	11p*
**23p**	CH261-191G17	23p*	**28d**	CH261-72A10	11p*

Homologies obtained both by whole chromosome painting and BAC probes are shown in figure 4.

Discussion

The karyotype of *S. tyrannus* obtained herein presented 2n=68, confirming data from a previous report [1]. We report slight differences in chromosome morphology however, due to the higher number of biarmed pairs (Table 1).

The results of comparative chromosome painting with whole chromosome probes of *G. gallus* showed a similar pattern to other birds of prey in the family Accipitridae, with a large reorganization of the syntenic groups homologous to the first five pairs of *G. gallus*. That is, each probe (GGA1 - GGA5) corresponded to at least two distinct pairs (Figure 3). The most extreme examples are the fission of GGA1 into six pairs in STY, and GGA3 into four distinct pairs. These results are congruent with other birds of prey,

considering that GGA1 can reveal syntenic segments in four pairs (*Gypaetus barbatus*, 2n=60) to seven pairs (*Nisaetus nipalensis orientalis* - NNI), while GGA3 is hybridized to four pairs in all species analyzed in this family. The exception is NNI where it hybridizes to only 2 pairs [11]. On the other hand, GGA6 - GGA10 are conserved syntenies, with only one signal for each pair.

All associations observed in the karyotype of STY based in its homology with *G. gallus* are represented in Figure 4. In general, 16 fissions and 13 fusions were detected, totalizing 29 rearrangements in the karyotype of STY when compared to *G. gallus*, with fissions occurring mainly in relation to the first five pairs of macrochromosomes and fusions involving mainly the microchromosomes. In the microchromosomes, chicken BAC probes showed that their syntenies were not disrupted by fission events as probes for proximal and distal regions were found hybridizing to the same pair in STY, except for GGA17, which produced signals in STY9 and STY24. However, all the identified BAC signals showed that each GGA microchromosome was fused to a STY segment homologous to either a GGA macro or microchromosome. This indicates that chromosomal fusions had an important role in reducing the diploid number in STY and other Accipitriformes. It is important to note that not all GGA microchromosomes are represented by chicken BACs, and hence other fusions must have occurred in this species to maintain 2n = 68.

The closest subspecies to *Spizaetus tyrannus* with chromosome painting data is the Japanese-Eagle (*Nisaetus nipalensis orientalis*), with 2n=66 [10]. Although geographically separated, they are morphologically similar, and until the last decade were classified as part of the same genus. Despite now being separated into distinct genera, molecular data support their close phylogenetic relationship [20]. Nevertheless, the comparative chromosome painting detects many differences. For instance, GGA1-9

probes produced signals in 21 pairs in STY, and 22 in NNI; the difference was due to an extra fission of GGA1 in NNI. Despite both species presenting three fusions involving the first nine pairs with microchromosomes (STY: pairs 4, 7 and 9; NNI: pairs 2, 4 and 9), none of them share the same GGA syntenic groups, and microchromosomes involved in NNI were not identified. Additionally, in both species GGA3 hybridizes to 4 pairs, however in STY all these segments are fused with microchromosomes (GGA: pairs 18, 19, 24 and 25), whereas in NNI only one segment of GGA3 is fused with a microchromosome (unidentified pair) [10].

Regarding the phylogenetic relationship of Aquilinae with other subfamilies within Accipitridae, although STY and NNI present some karyotypic similarities common to diurnal birds of prey, such as recurrent breakpoints mainly in relation to the GGA1-GGA5 pairs [10,11], we did not identify any synapomorphic associations which could represent ancestral characteristics for the Aquilinae [21-22]. Hence, while other subfamilies, such as Buteoninae and Harpiinae present well-established chromosomal signatures that allow the elaboration of their putative ancestral karyotypes [7], the available chromosome data indicate an absence of chromosomal signatures between STY and NNI, which can be explained by their significant geographic isolation, inhabiting opposite regions in the globe.

Conclusion

The present work is the first comparative chromosome mapping of a species in the genus *S. tyrannus* and has revealed substantial karyotypic reorganization common to birds of prey of the family Accipitridae. Together with *G. gallus* chromosome-specific probes for the larger pairs, chicken BACs were able to provide a more comprehensive

result with additional information on the organization of the *S. tyrannus* karyotype. There are many similarities with the *N. nipalensis orientalis,* including numerous fissions of the first five pairs homologous to GGA with only one less in STY (21 events against 22 in NNI), and three fusions involving homologues of GGA1-GGA9 chromosomes and microchromosomes, but with breakpoints that are not shared between these two species. For a broader analysis at the phylogenetic level, it would be necessary to have comparative mapping of other species of the genus *Spizaetus* so that an ancestral karyotype of this genus could be traced.

Methods

Samples and Chromosome Preparations

The experiments followed the standards approved by the Ethics Committee for the Use of Animals in Research (CEPAE-UFPA under number 170-13). We performed fibroblast cell cultures from skin biopsies and feather pulp of *Spizaetus tyrannus* (STY) obtained from two female individuals maintained in Zoos (Criadouro Gavião Real, Capitão Poço, Brazil), following the protocol of Sasaki et al. [23] with modifications. After tissue cleavage in Petri dishes, the samples were incubated with 1% type 1 collagenase (GIBCO) for 1 hour at 37°C for tissue dissociation. Metaphase chromosomes were obtained after incubation for one hour with Colcemid (0.05 µg/mL), hypotonic solution (KCl at 0.075 M) for 20 minutes and fixation in methanol/acetic acid (3:1). Karyotype analysis was performed using conventional staining with 5% Giemsa in 0.07 M phosphate buffer (pH 6.8) for 5 minutes, slides were analyzed using a 100× objective (Leica, CO, USA) and GenASIs software (ADS Biotec, Omaha, NE, USA).

GGA Probes and FISH Experiments

Two types of *Gallus gallus* probes were used: whole-chromosome-specific probes of the first 10 pairs, and bacterial artificial chromosomes (BACs) probes from 11 microchromosome pairs. Whole chromosome paints were developed and provided by the Cambridge Resource Center for Comparative Genomics (Cambridge, UK) using the Fluorescent Activated Cell Sorting (FACS) technique and labeled with biotin, fluorescein and/or digoxigenin (Roche Diagnostics, Mannheim, Germany), and detected with the addition of avidin-Cy3 (or Cy5) or anti-digoxigenin-rhodamin (Vector Laboratories, Burlingame, CA, USA). BAC clones ranged from 150,000 kb to 210,000 kb in size were selected from the CHORI-261 Chicken BAC library (Children's Hospital Oakland Research Institute, Oakland-USA), corresponding to sequences from the proximal and distal regions of the microchromosomes (each pair represented by two BACs, in a total of 22 BACs covering pairs 17 to 28, except for pair 20). Clones were produced following the protocol of the mini prep kit (Qiagen, Hilden, Germany) and labelled directly by fluorescein isothiocyanate (FITC) (green) or Texas Red (red) through Nick Translation (Roche, Mannheim, Germany).

Hybridization experiments followed standard procedures [7, 12]. Probes (1 µL labeled probe in 14 µL hybridization buffer) were denatured at 70ºC for 10 min and preannealed for 30 min at 37ºC. Hybridization mix was added on slides with chromosome preparations previously denatured at 70% formamide for 1 min and 20 s and dehydrated by serial ethanol dehydration (70%, 90% and 100%). Detection was performed using Avidin-Cy5 or anti-digoxygenin (Vector Laboratories, Burlingame, CA, USA). Slides were analyzed with an Olympus BX-61 epifluorescence microscope equipped with a cooled CCD camera and appropriate filters. Images were captured using SmartCapture3 (Digital Scientific UK).

Declarations

Ethics Approval: The experiments were carried out according to the ethical protocols approved by an ethics committee (CEUA—Federal University of Pará) under no. 170/2013 and SISBIO 68443-1.

Availability of Data and Materials: All data generated or analyzed during this study are included in this published article

Author Contributions: Conceptualization, C.C.A.L, I.O.F. and E.H.C.O.; Data curation and formal analysis, C.C.A.L., I.O.F., J.P. and E.H.C.O.; Investigation, C.C.A.L., I.O.F., Methodology, I.O.F., P.C.M.O., and J.P.; Project administration, E.H.C.O.; Funding acquisition, M.A.F.S., D.K.G. and E.H.C.O.; Validation, C.C.A.L., I.O.F. and E.H.C.O.; Writing (original draft), C.C.A.L., I.O.F. and E.H.C.O.; Writing (review and editing), D.K.G., P.C.M.O. and M.A.F.S.

Acknowledgements: We would like to thank all the staff of the Laboratório de Citogenômica e Mutagênese Ambiental (SAMAM, IEC) and Cambridge Resource Centre for Comparative Genomics for their technical support.

Conflict of interest: The authors declare that they have no conflict of interest

References

1. Tagliarini, M. M.; Nagamachi, C. Y.; Pieczarka, J. C. & De Oliveira, E. H. C. Description of two new karyotypes and cytotaxonomic considerations on Falconiformes. Brazilian J Ornit, 2007; 15(2): 167-172.

2. De Boer, L. E. M. The somatic chromosomes of 16 species of Falconiformes (Aves) and the karyological relationships of the order. Genética, 1990; 46: 77.

3. De Boer, L.E.M. The somatic chromosome complements of 16 species of Falconiformes (Aves) and the karyological relationships of the order. Genetica, 1976, 46: 77-113.

4. Santos, L. P. & Gunski, R. J. Revisão de dados citogenéticos sobre a avifauna brasileira. Brazilian J Ornith, 2006; 14: 35-45.

5.Kretschmer, R.; Ferguson-Smith, M.A.; de Oliveira,E.H.C. Karyotype Evolution in Birds: From Conventional Staining to Chromosome Painting. Genes, 2018; 9:181.

6. de Oliveira, E. H. C.; Habermann, F.; Lacerda, O.; Sbalqueiro, I. J.; Wienberg, J. & Muller, E. S. Chromosome reshuffling in birds of prey: the karyotypes of the world's largest eagle (Harpy eagle, *Harpia harpyja*) compared to that of the chicken (*Gallus gallus*). Chromosoma, 2005; 114: 338-343.

7. de Oliveira, E. H. C.; Tagliarini M. M.; Rissino J. D.; Pieczarka, J. C.; Nagamachi, C. Y.; O´Brien, P. C. M. & Ferguson-Smith, M. A. Reciprocal chromosome painting

between white hawk (*Leucopternis albicollis*) and chicken reveals extensive fusions and fissions during karyotype evolution of accipitridae (Aves, Falconiformes). Chromosome Res, 2010; 18: 349–355.

8. de Oliveira, E. H. C.; Tagliarini M. M.; Santos, M. S.; O´Brien, P. C. M. & Ferguson-Smith, M. A. Chromosome painting in three species of buteoninae: a cytogenetic signature reinforces the monophyly of south american species. Plos One, 2013; 8(7), 5–10.

9. Nanda I; Karl E; Volobouev V; Griffin DK; Schartl M; Schmid M. Extensive gross genomic rearrangements between chicken and Old World vultures (Falconiformes: Accipitridae). Cytogenet Genome Res, 2006; 112:286-295.

10. Nishida C, Ishijima J, Ishishita S, Yamada K, Griffin DK, Yamazaki T, et al. Karyotype reorganization with conserved genomic compartmentalization in dot-shaped microchromosomes in the Japanese mountain hawk-eagle (*Nisaetus nipalensis orientalis*, Accipitridae). Cytogenet Genome Res, 2013;141:284–94.

11. Nie, W.; O´Brien, P. C. M.; Fu, B.; Wang, J.; Su, W.; He, K.; Bed´Hom, B. et al. Multidirectional chromosome painting substantiates the occurrence of extensive genomic reshuffling within Accipitriformes. BMC Evolutionary Biology, 2015; 15:205.

12. O'Connor, R. E.; Kiazin, L.; Skinner, B.; Fonseka, G.; Joseph, S.; Jennings, R. et al. Patterns of microchromosome organization remain highly conserved throughout avian evolution. Chromosoma, 2018; 128: 21–29.

13. Waters, P. D.; Patel, H. R.; Ruíz-Herrera, A.; Álvarez-González, L.; Lister, N. C.; Simakov, O.et al. Microchromosomes are building blocks of bird, reptile and mammal chromosomes. BioRxiv [Preprint] 2021 bioRxiv [Posted 2021 July 07]. Available from: https://www.biorxiv.org/content/10.1101/2021.07.06.451394v1.

14. Furo, I.O.; Kretschmer, R.; O'Brien, P.C.M.; Pereira, J.; Garnero, A.D.V.; Gunski, R. et al. Chromosomal evolution in the phylogenetic context in Neotropical Psittacidae with emphasis on a species with high karyotypic reorganization (*Myiopsitta monachus*). Front. Genet. 2020, 11: 721.

15. Kretschmer, R.; de Souza, M.S.; Furo, I.d.O.; Romanov, M.N.; Gunski, R.J.; Garnero, A.d.V.; de Freitas, T.R.O.; de Oliveira, E.H.C.; O'Connor, R.E.; Griffin, D.K. Interspecies Chromosome Mapping in Caprimulgiformes, Piciformes, Suliformes, and Trogoniformes (Aves): Cytogenomic Insight into Microchromosome Organization and Karyotype Evolution in Birds. Cells, 2021, 10: 826.

16. Crooijmans, R.; Vrebalov, J.; Dijkhof, R; Van Der Poel, J. & Groenen, M. Two-dimensional screening of the Wageningen chicken BAC library. Mammalian Genome, 2000; 11(5): 360-363.

17. Lithgow, P. E.; O´Connor, R.; Smith, D.; Fonseka, G.; Al Mutery, A.; Rathje, C.; Frodsham, R et al. Novel tools for characterising inter and intra chromosomal rearrangements in avian microchromosomes. Chromosome Res, 2014; 22:85–97.

18. Rangel, T.F.L.V.; Diniz-Filho, J.A.F. Worldwide patterns in species richness of Falconiformes: Analytical null models, geometric constraints, and the mid-domain effect. Braz. J. Biol., 2004, 64: 299-308.

19. Degrandi T, M, Barcellos S, A, Costa A, L, Garnero A, D, V, Hass I, Gunski R, J: Introducing the Bird Chromosome Database: An Overview of Cytogenetic Studies in Birds. Cytogenet Genome Res 2020. doi: 10.1159/000507768.

20. Haring, E.; Kvaloy, K.; Gjershaug, J.; Rov, N. & Gamauf, A. Convergent evolution and paraphyly of the hawk-eagles of the genus *Spizaetus* (Aves, Accipitridae) – phylogenetic analyses based on mitochondrial markers. J Zool Syst Evol Res, 2007; 45(4): 353–365.

21. Romanov, M. N.; Farré, M.; Lithgow, P. E.; Fowler, K. E.; Skinner, B. M.; O'connor, R.; Fonseka, G.; Backström, N.; Matsuda, Y.; Nishida, C et al. Reconstruction of gross avian genome structure, organization and evolution suggests that the chicken lineage most closely resembles the dinosaur avian ancestor. BMC Genomics, 2014; 15:1060.

22. Zhang, G.; Li, C.; Li, Q.; Li, B.; Larkin, D. M.; Lee, C.; Storz, J. F. et al. Comparative genomics reveals insights into avian genome evolution and adaptation. Science, 2014; 346: 1311-1319.

23. Sasaki, M. et al. A Feather Pulp Culture Technique for Avian Chromosomes, with Notes on the Chromosomes of the Peafowl and the Ostrich. Experientia, 1968; 24(12):1292-1293.

Figure Legends

Figure 1- Metaphase (A) and karyotype (B) of *S. tyrannus* with diploid number 2n = 68, obtained with Giemsa conventional staining. The red arrows in (A) indicate the sex chromosomes. Scale bar: 5µm

Figure 2- Representative results of FISH experiments using *G. gallus* chromosome-specific probes corresponding to pairs GGA1 to GGA5 in *S. tyrannus* karyotype. Red and green signals represent probes labelled with Cy3 or FITC, respectively. Scale bar: 5µm

Figure 3- Representative results of hybridizations with some *G. gallus* BACs probes in the karyotype of *S.tyrannus*. (A1 and A1.1) Chicken BAC17 was the only one to hybridize

different chromosomes. Red signals represent probes labeled with Cy3, corresponding to the proximal region (p); Green signals represent probes labeled with FITC, corresponding to the distal region (d). Arrows indicate the signals. Scale bar: 5µm

Figure 4- Idiogram representing the homology between the *S. tyrannus* chromosomes and the macrochromosome chromosome-specific probes and microchromosomes BAC clones from *G. gallus*. Empty boxes mean no signal detected in those chromosomes with the set of probes used. BACs corresponding to pairs 20 and 22 (*) was not used or did not produce any detectable signals, respectively.

Figure 1 edited

Click here to access/download;Figure;1 - figure01-edited.tif

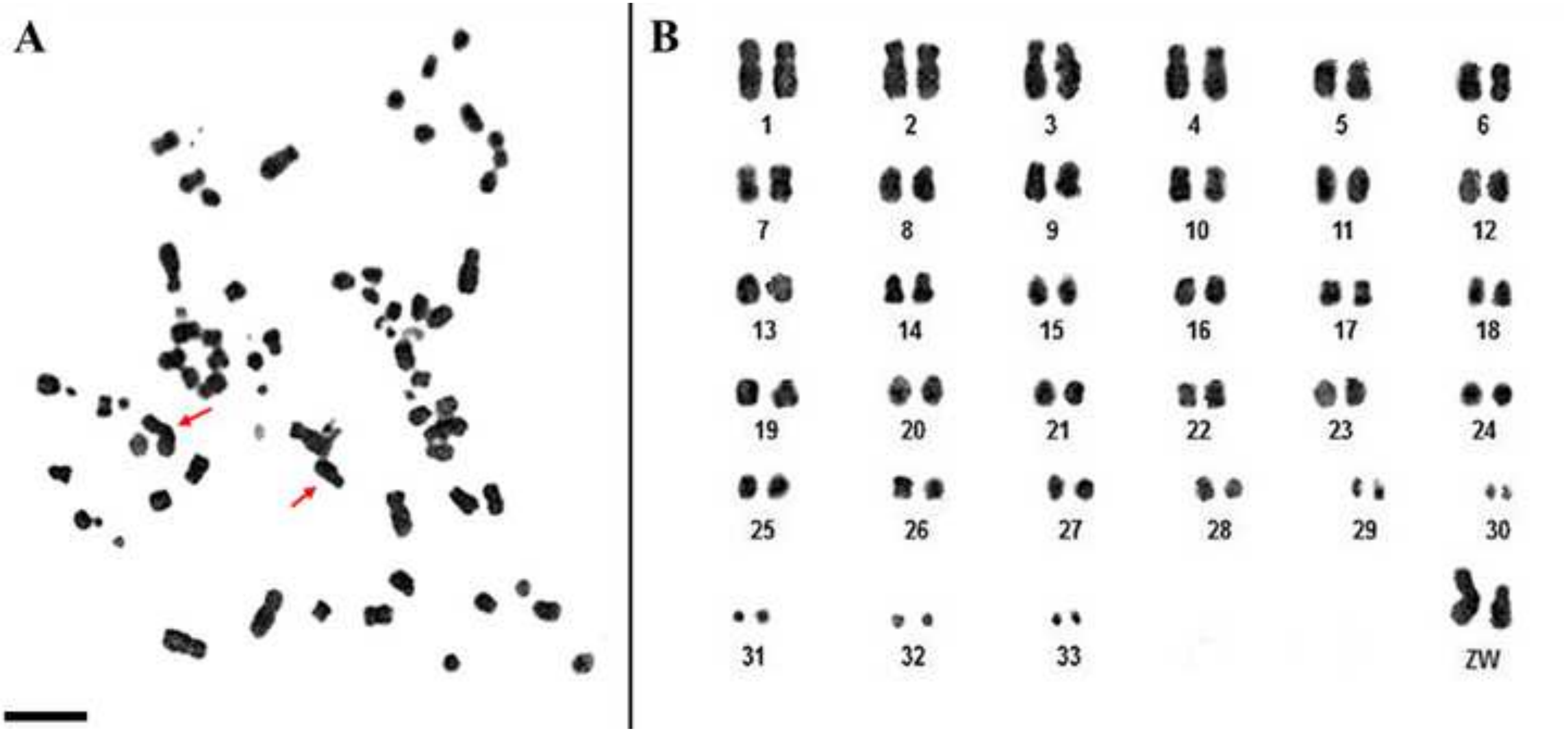

Figure 2 edited

Click here to access/download;Figure;2 - figure03-edited.tif

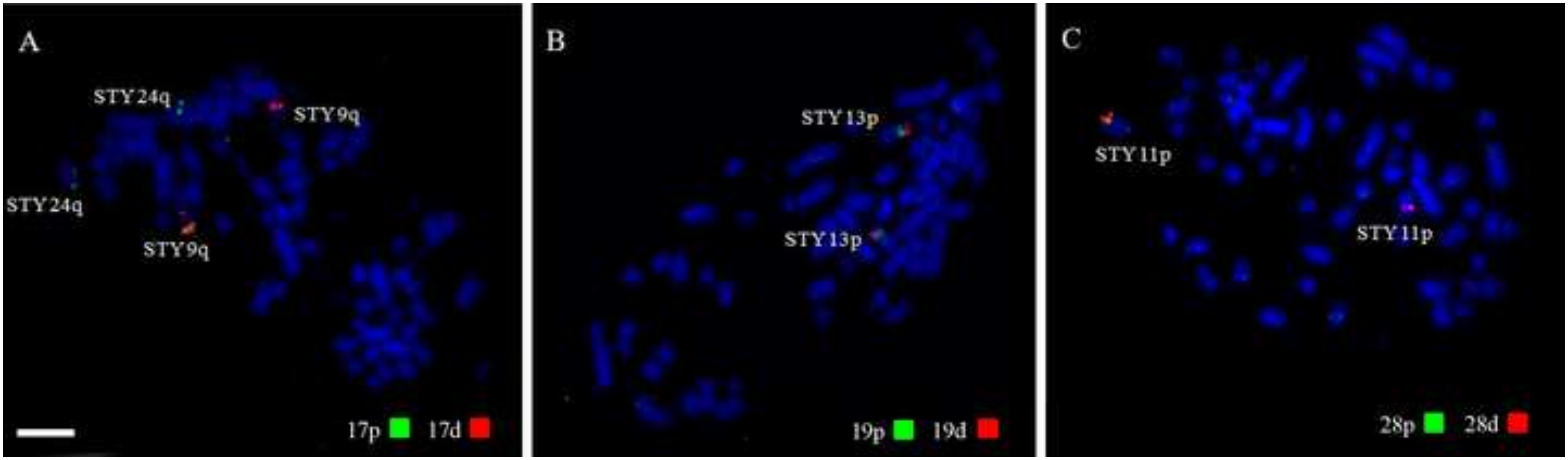

Figure 3 edited

Click here to access/download;Figure;3 - figure03-edited.tif

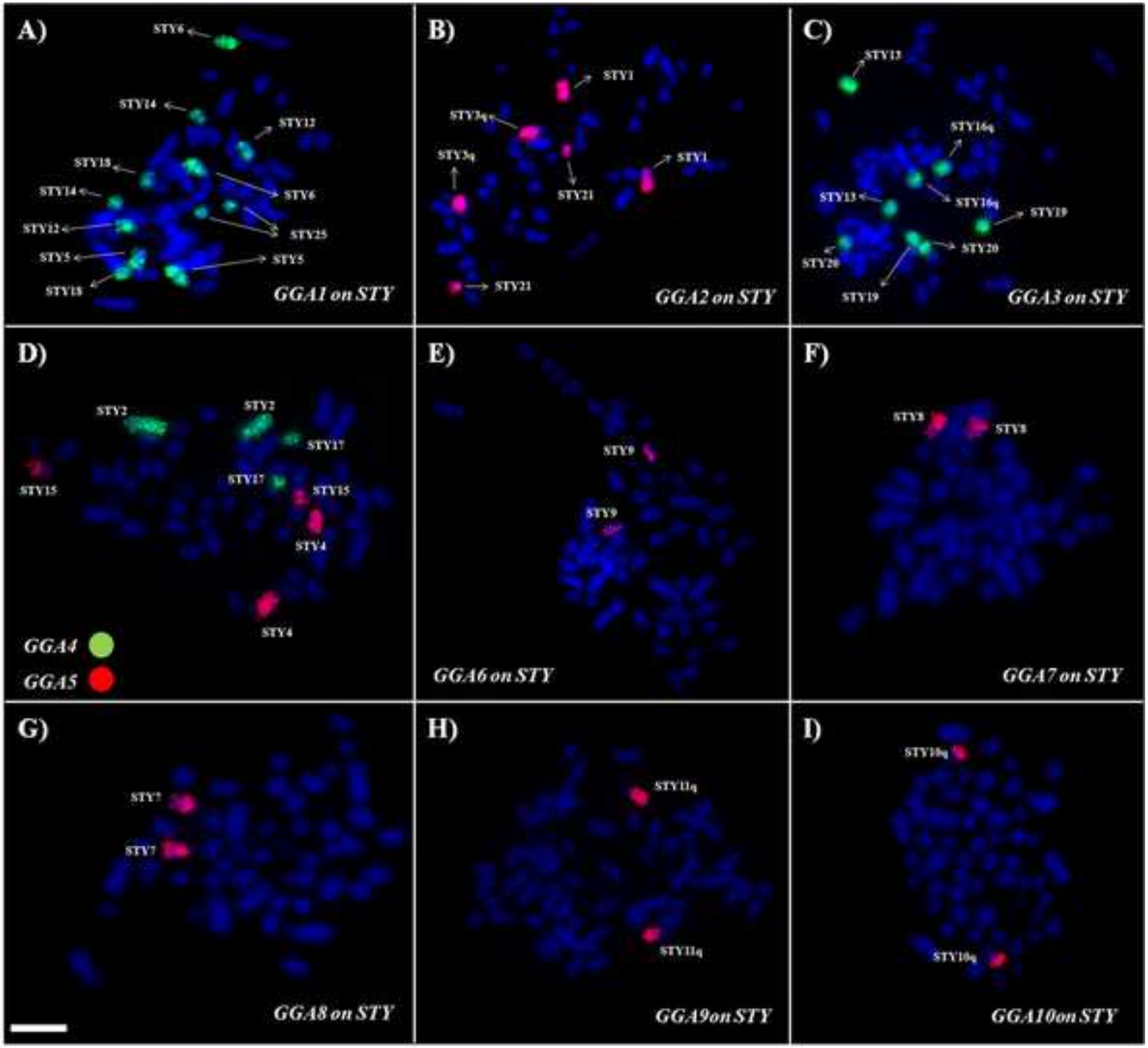

Figure 4

Click here to access/download;Figure;figure04.tif

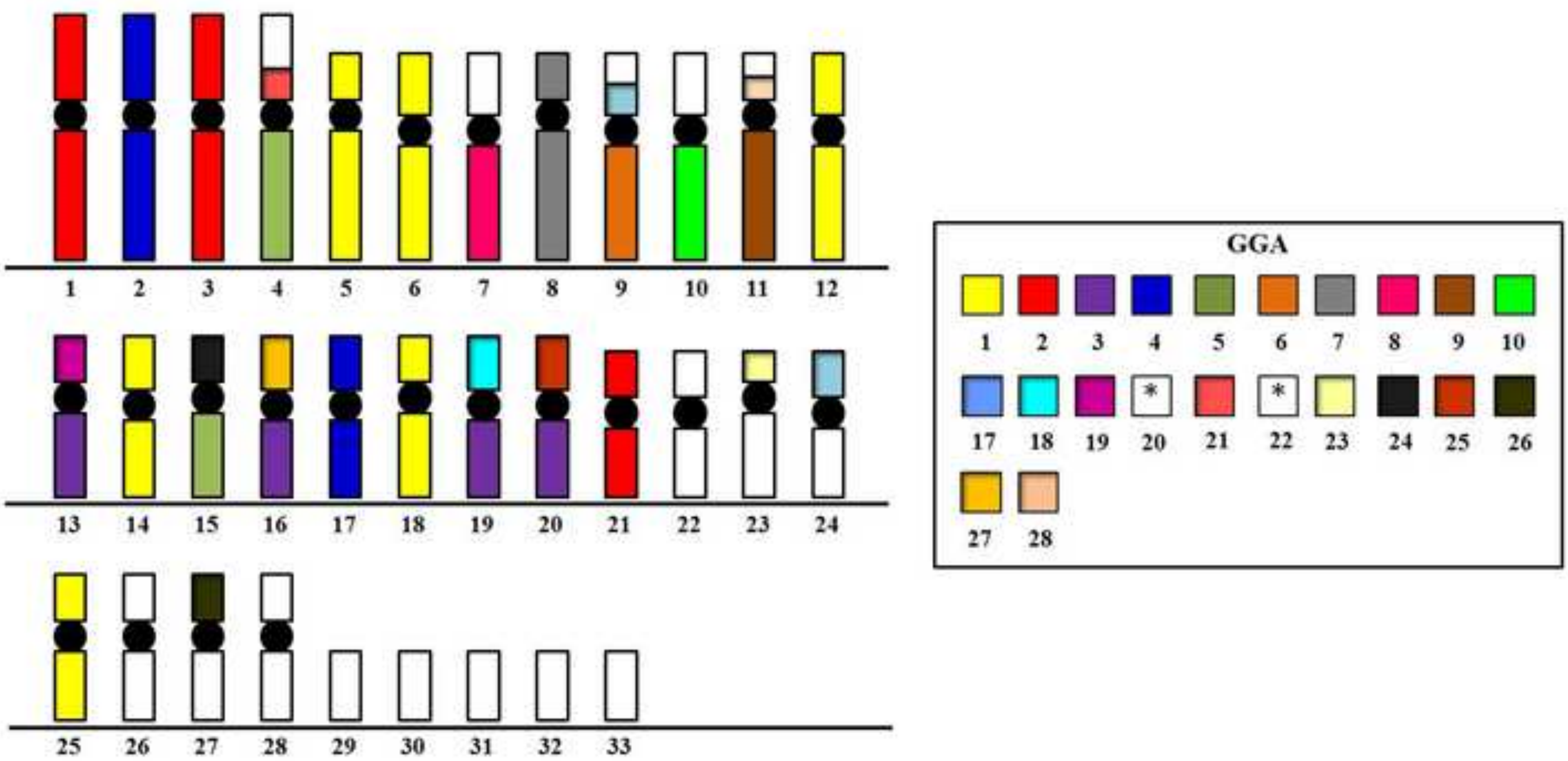

Comparative Chromosome Painting in the Black-Hawk-Eagle (*Spizaetus tyrannus*) and *Gallus gallus* with the use of Macro and Microchromosome Probes.

Short Title: Comparative Chromosome Painting in *Spizaetus tyrannus* and *Gallus gallus*

Carlos Augusto L Carvalho[1, 3], Ivanete de Oliveira Furo[1, 2, 3], Patricia C. M. O'Brien[43], Jorge Pereira J [54], Rebeca E. O'Connor [65], Darren Griffin[65], Malcolm A. Ferguson-Smith MA[43], Edivaldo Herculano C. de Oliveira[32,76] *

1 Programa de Pós-Graduação em Genética e Biologia Molecular, Universidade Federal do Pará, Belém, Pará 66075-110, Brazil

2 Universidade Federal Rural da Amazônia (UFRA) Laboratório de Reprodução Animal (LABRAC), Parauapebas, PA, Brazil

32 Laboratório de Cultura de Tecidos e CitogenéticaCitogenômica e Mutagênese Ambiental, SAMAM, Instituto Evandro Chagas, Ananindeua, Pará 67030-000, Brazil

43 Cambridge Resource Centre for Comparative Genomics, Cambridge CB3 0ES, UK

54 4 Animal and Veterinary Research Center, Universidade de Trá-os-Montes e Alto douro, Vila Real, Portugal

65 School of Biosciences, University of Kent, Canterbury CT2 7NJ, UK

76 Faculdade de Ciências Naturais, Instituto de Ciências Exatas e Naturais, Universidade Federal do Pará, Belém, Pará 66075-110, Brazil

Corresponding Author:

Formatted: Font: 12 pt

Formatted: English (United Kingdom)

Formatted: Font: 12 pt

Formatted: Justified

Formatted: Don't adjust space between Latin and Asian text, Don't adjust space between Asian text and numbers

Formatted: Font: Not Italic, Portuguese (Brazil)

Formatted: Font: Not Italic

Formatted: Font: Not Italic, Portuguese (Brazil)

Formatted: Font: (Default) TimesNewRomanPS-ItalicMT, Font color: Auto, Pattern: Clear

Formatted: Space After: 0 pt

***Dr. Edivaldo Herculano C. de Oliveira**

ehco@ufpa.br, edivaldooliveira@iec.gov.br

Abstract

Although mMThe most birds shows karyotypes with diploid number around 2n=80, consist ofwith few macrochromosomes and many microchromosomes pairs, however some groups, such as the AAccipitriformes, are characterized by a large karyotypic reorganization, which resulted in complements with low diploid numbers, and a smaller number of microchromosomal pairs when compared to the karyotypes of most other birds, with diploid numbers around 2n=80 and many pairs of microchromosomes. Among Accipitriformes, the Accipitridae family is the most diverse and is currently divided into 14 subfamilies, includingincludes the subfamily Aquilinae, composed of medium to large sized species. , including thean example is BlacktThe Black-Hawk-Eagle (*Spizaetus tyrannus* - STY), found in South America, is a member of this subfamily. Available chromosome data for this species includes only conventional staining. HereHence, in order to provide additional information onre, aiming understanding the aspects of karyotypicce evolution process within theis group Aquilinae Subfamily, we presenting in this study theperformed comparative chromosome mappingpainting chromosome homologies between *S. tyrannus* this species and the *chicken Gallus gallus* (GGA) using comparative chromosome painting. Our results revealed that at least 29 fission-fusion events were observed in the STY karyotype, based on homology with GGA. The majority of fFissions occuredoccurred mainly in segments syntenic groups homologous to GGA1-GGA5, whereas the majority of the GGA microchromosomes remained conserved

Formatted: Font: Italic

andwere found fused to other chromosomal elements in STY. Comparison withWhen compared to hybridization pattern of the Japanese-Mountain-Eagle (*Nisaetus nipalensis orientalis*), the only close relative of *S. tyrannus* analyzed by comparative -chromosome painting so far, we observed that there areshowed many some similarities, but also and differences between these species that, includingconcerning numerous fissions and fusions events of the first five pairs homologous to GGA. Therefore, conclusions about karyotype evolution in Aquilinae require additional painting studies with only one less in STY (21 events against 22 in NNI), and three fusions involving homologues of GGA1-GGA9 chromosomes and microchromosomes, but with breakpoints that are not shared between these two species. However, for a phylogenetic analyze within of the subfamily Aquilinae would be necessary comparative mapping of other species of the genus *Spizaetus* so that an ancestral karyotype of this subfamily could be traced.

Key Words: Aquilinae, Chromosome painting, BACs, Microchromosomes

Introduction

ThUsually, bird ge Genome is organized in karyotypes s of birds, consisting of few macrochromosomes and many tiny microchromosomes. However, there are some excetions. For instance, eExcluding the New World vultures (Cathartidae), which show

similar karyotypes to the putative avian ancestral karyotype with diploid number around 2n= 80, including 10 pairs of macrochromosomes and 30 pairs of microchromosomes [1], species belonging to the Order Accipitriformes present with an unusual chromosomal diversity. They have smaller lower diploid numbers, 2n = 54-68, and a reduction of microchromosomes to between 4 and 8 pairs, due mainly to several chromosomesfusions involving these small elements rearrangements that, occurred during their divergence [2-4].

In generally, the studies aboutfocusing on the chromosome evolution in birds are Accipitriformes were mainly based in comparative chromosome painting using chicken whole chromosome paintsprobes of the chicken (*Gallus gallus* – GGA, 2n=78) the most common chromosome probe used in birds, due to the similarity of the genome organizationkaryotype of this species with the proposed putative avian ancestral karyotype (PAK) of this species as the ancestral of Aves [5]. According to these studiedThe use of this methodology in species of birds of prey has revealed that, despite the lower diploid numbers observed in this group, , comparative chromosome painting studies using whole chromosome paints of the chicken (*Gallus gallus* – GGA), thisthe the large karyotype reorganization in Accipitriformes included several manymultiple fissions in the macrochromosome macrochromosome pairs homologous to GGA1-GGA5. The reduction of the chromosome number would be due to the concomitant occurrence of and largeseveral f fusion events involving , while microchromosomes were largely involved in fusion events [65-110].

The mMicrochromosomes in birds karyotype are gene rich elements, and and highly conserved, some genome comparative analysies have shown considerabletheir conservation as syntenic groups among distantly related bird groups [12-13]. In fact, rearrangements involving microchromosomes were detected only in in fewsome order: ,

Formatted: Font: (Default) Times New Roman, 12 pt, English (United Kingdom)

Formatted: Font: (Default) Times New Roman, 12 pt

Formatted: Font: (Default) Times New Roman, 12 pt, English (United Kingdom)

Formatted: Font: (Default) Times New Roman, 12 pt

Formatted: Font: (Default) Times New Roman, 12 pt

Formatted: Font: (Default) Times New Roman, 12 pt, English (United Kingdom)

Formatted: Font: (Default) Times New Roman, 12 pt, English (United Kingdom)

Formatted: Font: (Default) Times New Roman, 12 pt, English (United Kingdom)

Formatted: Font: (Default) Times New Roman, 12 pt, English (United Kingdom)

Formatted: Font: (Default) Times New Roman, 12 pt, English (United Kingdom)

Formatted: Font: (Default) Times New Roman, 12 pt, English (United Kingdom)

~~such as~~ Psittaciformes, Cuculiformes and the Accipitriformes ~~were detected rearrangements involving these elements~~ [13-15].

Due to difficulties ~~involving~~ of the isolation of individual microchromosome pairs by flow cytometry for specific probe production, most data concerning microchromosomes were obtained ~~have been obtained using~~by the use of pools of microchromosomes, i.e., chromosome paints that recognize more than one pair. Therefore, improved identification of chromosome pairs involved in rearrangements is a priority if we are to achieve a more definitive analysis and identify synapomorphies based on chromosome characters [16~~1~~-17~~2~~].

Formatted: Adjust space between Latin and Asian text, Adjust space between Asian text and numbers

Currently, the order Accipitriformes is composed of four families, of which ~~the~~ Accipitridae ~~family being~~is the most diverse, with approximately 230 species distributed in 14 subfamilies~~ in quantity of species, within this family is the subfamily Aquilinae~~ [18]. Among them, the subfamily Aquilinae includes medium and large species, distributed globally, usually known as booted eagle. Usually, ten genera are found within Aquilinae, of which only four have their diploid numbers defined, ranging from 2n=66 to 68 [19].

The Black-Hawk-Eagle (*Spizaetus tyrannus*-STY) is a representative of this subfamily, ~~belongs to this subfamily. This species~~ ~~belongs to the subfamily Aquilinae (Accipitriformes, Accipitridae), and is~~ found in South and Central Americas~~n~~, from southern Mexico down to Argentina [18~~9~~].~~(Sick, 1997; Mikich & Bernils, 2004).~~ Considering that t~~T~~he only chromosomal analysis of *S. tyrannus* to date was based on conventional staining, revealing a karyotype within the ~~Accipitridae~~ Aquilinae standard, with 2n=68 ~~and only four pairs of microchromosomes~~ [1], the aim of this study was to present ~~.~~the cytogenetic mapping of *S. tyrannus* by comparative painting. In addition to

Commented [if1]: Falta acrescentar essas referências.

whole-chromosome paints of *Gallus gallus* (GGA), we used BAC probes from GGA clones that identified 11 individual pairs of microchromosomes. The results were compared to *Nisaetus nipalensis orientalis*- NNI (2n=66) [10], also from the subfamily Aquilinae, in order do identify chromosomal rearrangements related to karyotype evolution in this group.

Thus,is study aimed understanding the karyotype evolution within of the subfamily Aquilinae, we presenteds the cytogenetic mapping of *S. tyrannus*a species of the genus *Spizaetus* by comparative painting, we also compared this species to the *Nisaetus nipalensis orientalis*- NNI (2n=66) [10], the only close relative. In addition Additionally, to whole-chromosome paints of *Gallus gallus* (GGA), we used bacterial artificial chromosomes (BACs) BAC probes from *GGA G. gallus* clones that identified corresponded to 11 individual pairs of microchromosomes were used aiming identify possible rearrangements that could help to understand the role of microchromosomes in the karyotype evolution of *S. tyrannus*..

Formatted: Font: Italic

Results

Karyotype description

Formatted: Font: Italic

The karyotype of *Spizaetus tyrannus* presenteds a diploid number 2n=68, consisting of 21 meta or submetacentric pairs (pairs 1-4, 6-7, 9-10, 12, 14, 16-17, 19-22, 24-298 and the sex chromosomes), 7 seven acrocentric (pairs 5, 8, 11, 13, 15, 18 and 23), and 4four4 pairs of microchromosomes (pairs 2930-33). The Z chromosome is a large

metacentric, with size between pairs 3 or 4, while the W chromosome is an average submetacentric, similar in size to pairs 8 or 9 (Figure 1). In table 1, we ~~We~~ reported ~~at this study~~ some differences in chromosome morphology of *S.tyrannus* described by Tagliarini et al., [1] (Table 1).

Table 1- Karyotype of *S. tyrannus* described by Tagliarini et al., 2006 and at this study. (Metacentric: M; Submetacentric: SM; Subtelocentric: ST; Acrocentric: AC)

**Pairs**	**This study**	**Tagliarini et al., 2007**	**Pairs**	**This study**	**Tagliarini et al., 2007**
**Chr 1.**	SM	SM	Chr 18.	AC	AC
**Chr 2.**	SM	SM	Chr 19.	SM	SM
**Chr 3.**	SM	SM	Chr 20.	SM	SM
**Chr 4.**	SM	SM	Chr 21.	SM	SM
**Chr 5.**	AC	SM	Chr 22.	SM	SM
**Chr 6.**	SM	ST	Chr 23.	AC	AC
**Chr 7.**	SM	SM	Chr 24.	SM	AC
**Chr 8.**	AC	ST	Chr 25.	SM	AC
**Chr 9.**	SM	SM	Chr 26.	SM	AC
**Chr 10.**	SM	ST	Chr 27.	SM	SM
**Chr 11.**	AC	SM	Chr 28.	SM	AC
**Chr 12.**	SM	SM	Chr 29.	SM	SM
**Chr 13.**	AC	SM	Chr 30.	Micro	Micro
**Chr 14.**	SM	AC	Chr 31.	Micro	Micro
**Chr 15.**	AC	AC	Chr 32.	Micro	Micro
**Chr 16.**	SM	ST	Chr 33.	Micro	Micro
**Chr 17.**	SM	ST	Chr ZW.	M and SM	M and SM

Comparative Chromosome painting

~~*GGA*~~ *G. gallus* probes used in the fluorescent *in situ* hybridization (FISH) experiments produced reproducible results. Hybridizations with chromosome-specific probes for the first ten pairs of GGA produced 22 signals, with the first five pairs producing multiple signals, ranging from 2 to 6 signals (Figure 2). For instance, GGA1 probe painted six distinct pairs in the *S. tyrannus* ~~STY~~ karyotype (pairs 5, 6, 12, 14, 18, 25), while the probes GGA6-10 pairs showed only one signal each. Table 2~~1~~ details the distribution of the signals produced by GGA whole specific probes in the karyotype of ~~*Spizaetus*~~ *S. tyrannus*.

Probe	STY Chromosomes	Probe	STY Chromosomes
GGA1	(5, 6, 12, 14, 18, 25)	GGA6	9
GGA2	(1, 3q, 21)	GGA7	8
GGA3	(13, 16q, 19, 20)	GGA8	7
GGA4	(2, 17)	GGA9	11q
GGA5	(4, 15q)	GGA10	10q

Table 2~~1~~ - Results of hybridizations with ~~*Gallus*~~ *G. gallus* probes showing the homology between GGA probes in the karyotype of ~~*Spizaetus*~~ *S. tyrannus* (STY).

A total of 19 out of 22 ~~*Gallus*~~ *G. gallus* BAC clones produced results. Both BACs from the GGA22 chromosome did not produce any detectable signal, as well as one of the BACs from GGA21. Among the 19 probes that gave good quality results, both proximal (BACp) and distal (BACd) referring to 8 pairs, were found in the same segment

Formatted: Font: Italic
Formatted: Font: Italic
Formatted: Font: Italic
Formatted: Line spacing: Double
Formatted: Font: Bold
Formatted: Font: Bold
Formatted: Font: Bold
Formatted: Font: Bold
Formatted: Font: Not Bold
Formatted: Font: Not Bold
Formatted: Font: Not Bold
Formatted: Font: Not Bold
Formatted: Font: Not Bold
Formatted: Font: Not Bold
Formatted: English (United Kingdom)
Formatted: Font: Not Bold
Formatted: Font: Not Bold
Formatted: English (United Kingdom)
Formatted: Font: Not Bold
Formatted: Font: Not Bold
Formatted: English (United Kingdom)
Formatted: English (United Kingdom)
Formatted: English (United Kingdom)
Formatted: English (United Kingdom)
Formatted: Indent: First line: 0"

in the STY karyotype. However, BACs corresponding to GGA17 hybridized to two different pairs - BAC17p marked STY 9q, while BAC17d marked STY 24q. (Figure 3). All Chicken BACs and their respective homology in the karyotype of Spizaetus S. *tyrannus* are summarized in Table 32.

Table 32 : Summary of the results of experiments using GGA BACs on the karyotype of *Spizaetus S. tyrannus* (*=BACs marking the same segment in STY karyotype; p=proximal region; d=distal region).

GGA	BAC ID	STY	GGA	BAC ID	STY
**17p**	CH261-113A7	9q	**23d**	CH261-90K11	23p*
**17d**	CH261-42P16	24q	**24p**	CH261-103F4	15p*
**18p**	CH261-60N6	19p*	**24d**	CH261-65O4	15p*
**18d**	CH261-72B18	19p*	**25p**	CH261-59C21	20p*
**19p**	CH261-10F1	13p*	**25d**	CH261-127K7	20p*
**19d**	CH261-50H12	13p*	**26p**	CH261-186M13	27p*
**21p**	CH261-83I20	No signal	**26d**	CH261-170L23	27p*
**21d**	CH261-122K8	4p	**27p**	CH261-66M16	16p*
**22p**	CH261-40J9	No signal	**27d**	CH261-28L10	16p*
**22d**	CH261-18G17	No signal	**28p**	CH261-64A15	11p*
**23p**	CH261-191G17	23p*	**28d**	CH261-72A10	11p*

Homologies obtained both by whole chromosome painting and BAC probes are shown in figure 4.

Discussion

The karyotype of *Spizaetus S. tyrannus* obtained herein presented 2n=68, confirming data from a previous report [1]. We report slight differences in chromosome morphology however, due to the higher number of biarmed pairs (Table 1).

The results of comparative chromosome painting with whole chromosome probes of ~~*Gallus*~~ *G. gallus* showed a similar pattern to other birds of prey in the family Accipitridae, with a large reorganization of the syntenic groups homologous to the first five pairs of *G. gallus*~~*allus*~~. That is, each probe (GGA1 - GGA5) corresponded to at least two distinct pairs (Figure 3). The most extreme examples are the fission of GGA1 into six pairs in STY, and GGA3 into ~~three~~ four distinct pairs. These results are congruent with other birds of prey, considering that GGA1 can reveal syntenic segments in four pairs (*Gypaetus barbatus*, 2n=60) to seven pairs (*Nisaetus nipalensis orientalis* - NNI), while GGA3 is hybridized to four pairs in all species analyzed in this family. The exception is NNI where it hybridizes to only 2 pairs [11~~0~~]. On the other hand, GGA6 - GGA10 are conserved syntenies, with only one signal for each pair.

All associations observed in the karyotype of STY based in its homology with ~~*Gallus*~~ *G. gallus* are represented in Figure 4. In general, 16 fissions and 13 fusions were detected, totalizing 29 rearrangements in the karyotype of STY when compared to ~~*Gallus*~~*G. gallus*, with fissions occurring mainly in relation to the first five pairs of macrochromosomes and fusions involving mainly the microchromosomes. In the microchromosomes, chicken BAC probes showed that their syntenies were not disrupted by fission events as probes for proximal and distal regions were found hybridizing to the same pair in STY. However, all the identified BAC signals showed that each GGA microchromosome was fused to a STY segment homologous to either a GGA macro or microchromosome. This indicates that chromosomal fusion led to the lower diploid number in STY and other Accipitriformes. Despite the numerous fissions observed, it is important to note that not all GGA microchromosomes are represented by chicken BACs, and hence other fusions must have occurred in this species to maintain a 2n = 68.

The closest subspecies to *Spizaetus tyrannus* with chromosome painting data is the ~~–~~Japanese-Eagle (*Nisaetus nipalensis orientalis*) [~~9~~10]. Although geographically separated, they are morphologically similar, and until the last decade were classified as part of the same genus. Despite now being separated into distinct genera, molecular data support their close phylogenetic relationship [~~14~~20]. Nevertheless, the comparative chromosome painting detects many differences. For instance, GGA1-9 probes produced signals in 21 pairs in STY, and 22 in NNI; the difference was due to an extra fission of GGA1 in NNI. Despite both species presenting three fusions involving the first nine pairs with microchromosomes (STY: pairs 4, 7 and 9; NNI: pairs 2, 4 and 9), none of them share the same GGA syntenic groups, and microchromosomes involved in NNI were not identified. Additionally, in both species GGA3 hybridizes to 4 pairs, however in STY all these segments are fused with microchromosomes (GGA: pairs 18, 19, 24 and 25), whereas in NNI only one segment of GGA3 is fused with a microchromosome (unidentified pair) [~~9~~10].

Another point relates to the difference in diploid number of *S. tyrannus* and *N. nipalensis orientalis* ~~*N. nipalensis*~~ (2n = 66 in NNI and 2n = 68 in STY). This can be explained by a centric fusion of two pairs of microchromosomes (pairs not identified) in the NNI7 chromosome resulting in one pair of microchromosomes less in the karyotype of *N. nipalensis orientalis* ~~*N. nipalensis*~~ [10~~9~~]. ~~These results show that they are morphologically similar species that until the last decade were part of the same genus [14].~~

Moreover, despite STY and NNI presenting some karyotypic similarities common to diurnal birds of prey such as recurrent breakpoints mainly in relation to the GGA1-GGA5 pairs [10~~9~~,11~~0~~], we did not identify any synapomorphic associations which could represent ancestral characteristics for the Aquilinae [~~15,16~~21-22]. Hence, while other

subfamilies, such as Buteoninae and Harpiinae present well-established chromosomal signatures that allow the elaboration of their putative ancestral karyotypes [76], the available chromosome data indicate an absence of chromosomal signatures between STY and NNI, which can be explained by their significant geographic isolation, inhabiting opposite regions in the globe.

Formatted: Indent: First line: 0"

Conclusion

Formatted: Justified

The present work is the first comparative chromosome mapping of a species in the genus ~~*Spizaetus*~~ *S. tyrannus* and has revealed substantial karyotypic reorganization common to birds of prey of the family Accipitridae. Together with ~~*Gallus*~~ *G. gallus* chromosome-specific probes for the larger pairs, chicken BACs were able to provide a more comprehensive result with additional information on the organization of the *S. tyrannus* ~~Black-Hawk-Eagle~~ karyotype. There are many similarities with the *N. nipalensis orientalis*~~Japanese Eagle~~, including numerous fissions of the first five pairs homologous to GGA with only one less in STY (21 events against 22 in NNI), and three fusions involving homologues of GGA1-GGA9 chromosomes and microchromosomes, but with breakpoints that are not shared between these two species. For a broader analysis at the phylogenetic level, it would be necessary to have comparative mapping of other species of the genus *Spizaetus* so that an ancestral karyotype of this genus could be traced. Furthermore, the new set of chromosome probes of *S. tyrannus* should be helpful in further studies of the phylogenetic relationships in Accipitriformes.

Methods

Samples and Chromosome Preparations

The experiments followed the standards approved by the Ethics Committee for the Use of Animals in Research (CEPAE-UFPA under number 170-13). We performed fibroblast cultures from skin biopsies and feather pulp of *Spizaetus tyrannus* (STY) obtained from two female individuals maintained in Zoos (Criadouro Gavião Real, Capitão Poço, Brazil), following the protocol of Sasaki et al. [2~~1~~3] with modifications. After tissue cleavage in Petri dishes, the samples were incubated with 1% type 1 collagenase (GIBCO) for 1 hour at 37°C for tissue dissociation. Metaphase chromosomes were obtained after incubation for one hour with Colcemid (0.05 µg/mL), hypotonic solution (KCl at 0.075 M) for 30 minutes and fixation in methanol/acetic acid (3:1). Karyotype analysis was performed using conventional staining with 5% Giemsa in 0.07 M phosphate buffer (pH 6.8) for 5 minutes, slides were analyzed using a 100× objective (Leica, CO, USA) and GenASIs software (ADS Biotec, Omaha, NE, USA).

GGA Probes and FISH Experiments

Two types of *Gallus gallus* probes were used: whole-chromosome-specific probes of the first 10 pairs, and ~~chromosome-specific~~ bacterial artificial chromosomes (BACs)~~BAC~~ probes from 11 microchromosome pairs. Whole chromosome paints were developed and provided by the Cambridge Resource Center for Comparative Genomics (Cambridge, UK) using the Fluorescent Activated Cell Sorting (FACS) technique and labeled with biotin, fluorescein and/or digoxigenin (Roche Diagnostics, Mannheim, Germany), and detected with the addition of avidin-Cy3 (or Cy5) or anti-digoxigenin-

rhodamin (Vector Laboratories, Burlingame, CA, USA). BAC clones ranged from 150,000 kb to 210,000 kb in size were selected from the CHORI-261 Chicken BAC library (Children's Hospital Oakland Research Institute, Oakland-USA), corresponding to sequences from the proximal and distal regions of the microchromosomes (each pair represented by two BACs, in a total of 22 BACs covering 11 chromosome pairs). Clones were produced following the protocol of the mini prep kit (Qiagen, Hilden, Germany) and labelled directly by fluorescein isothiocyanate (FITC) FTIC (green) or Texas Red (red) through Nick Translation (Roche, Mannheim, Germany).

Hybridization experiments followed standard procedures (de Oliveira et al., 2010[7]; O´Connor et al., [12]2018). The probes (1 µL labeled probe in 14 µL hybridization buffer) were denatured in 70ºC for 10 min and preannealed for 30 min at 37ºC, then the hybridization solution was added on slides with chromosome preparations previously desnatured at 70% formamide for 1 min and 20 s and dehydrated by serial ethanol dehydration (70%, 90% and 100%). The detection by Avidin-Cy5 or anti-digoxygenin (Vector Laboratories, Burlingame, CA, USA). Slides were analyzed with an Olympus BX-61 epifluorescence microscope equipped with a cooled CCD camera and appropriate filters. Images were captured using SmartCapture3 (Digital Scientific UK).

Formatted: Font: (Default) Times New Roman, 12 pt, English (United Kingdom)

Formatted: Line spacing: 1.5 lines, Don't adjust space between Latin and Asian text, Don't adjust space between Asian text and numbers

Formatted: Font: (Default) Times New Roman, 12 pt

Formatted: Font: (Default) Times New Roman, 12 pt

Formatted: Font: (Default) Times New Roman, 12 pt

Formatted: Font: (Default) Times New Roman, 12 pt

Formatted: Font: (Default) Times New Roman, 12 pt

Formatted: Font: (Default) Times New Roman, 12 pt

Formatted: Font: (Default) Times New Roman, 12 pt

Formatted: Font: (Default) Times New Roman, 12 pt

Formatted: Font: (Default) Times New Roman, 12 pt

Formatted: Font: (Default) Times New Roman, 12 pt

Formatted: Font: (Default) Times New Roman, 12 pt

Formatted: Font color: Black, English (United Kingdom)

Declarations

Ethics Approval: The experiments were carried out according to the ethical protocols approved by an ethics committee (CEUA—Federal University of Pará) under no. 170/2013 and SISBIO 68443-1.

Availability of Data and Materials: All data generated or analyzed during this study are included in this published article

Author Contributions: Conceptualization, C.C.A.L, I.O.F. and E.H.C.O.; Data curation and formal analysis, C.C.A.L., I.O.F., J.P. and E.H.C.O.; Investigation, C.C.A.L., I.O.F., Methodology, I.O.F., P.C.M.O., and J.P.; Project administration, E.H.C.O.; Funding acquisition, M.A.F.S., D.K.G. and E.H.C.O.; Validation, C.C.A.L., I.O.F. and E.H.C.O.; Writing (original draft), C.C.A.L., I.O.F. and E.H.C.O.; Writing (review and editing), D.K.G., P.C.M.O. and M.A.F.S.

Funding: This research was partially funded by a grant to EHCO from CNPq (307382/2019-2) and to MAFS from the Wellcome Trust in support of the Cambridge Resource Centre for Comparative Genomics and by the Biotechnology and Biological

Formatted: Don't adjust space between Latin and Asian text, Don't adjust space between Asian text and numbers

Sciences Research Council (BB/K008161/1) to the University of Kent, also by UIDB/CVT/00772/2020 funded by the Fundação para Ciência e Tecnologia (FCT)..

Formatted: Font: (Default) Times New Roman, 12 pt

Formatted: English (United Kingdom)

Formatted: Justified

Acknowledgements: We would like to thank all the staff of the Laboratório de Cultura de Tecidos e Citogenética (SAMAM, IEC) and Cambridge Resource Centre for Comparative Genomics for their technical support.

Conflict of interest: The authors declare that they have no conflict of interest

References

1. Tagliarini, M. M.; Nagamachi, C. Y.; Pieczarka, J. C. & De Oliveira, E. H. C. Description of two new karyotypes and cytotaxonomic considerations on Falconiformes. Brazilian J Ornit, 2007; 15(2): 167-172.

2. De Boer, L. E. M. The somatic chromosomes of 16 species of Falconiformes (Aves) and the karyological relationships of the order. Genética, 1990; 46: 77.

Formatted: English (United Kingdom)

3. De Boer, L.E.M. The somatic chromosome complements of 16 species of Falconiformes (Aves) and the karyological relationships of the order. Genetica, 1976, 46: 77-113.

Formatted: Portuguese (Brazil)

~~3. Bed'Hom B. *Etude des Caryotypes Atypiques des Accipitridae (Aves, Falconiformes) par Cytogènètique Classique et Mole¤ culaire, et Modèlisation de leur Evolution.* PhD thesis, University of Tours, 1999.~~

4. Santos, L. P. & Gunski, R. J. Revisão de dados citogenéticos sobre a avifauna brasileira. Brazilian J Ornith, 2006; 14: 35-45.

5.Kretschmer, R.; Ferguson-Smith, M.A.; de Oliveira,E.H.C. Karyotype Evolution in Birds: From Conventional Staining to Chromosome Painting. Genes, 2018; 9:181.

65. de Oliveira, E. H. C.; Habermann, F.; Lacerda, O.; Sbalqueiro, I. J.; Wienberg, J. & Muller, E. S. Chromosome reshuffling in birds of prey: the karyotypes of the world's largest eagle (Harpy eagle, *Harpia harpyja*) compared to that of the chicken (*Gallus gallus*). Chromosoma, 2005; 114: 338-343.

76. de Oliveira, E. H. C.; Tagliarini M. M.; Rissino J. D.; Pieczarka, J. C.; Nagamachi, C. Y.; O´Brien, P. C. M. & Ferguson-Smith, M. A. Reciprocal chromosome painting between white hawk (*Leucopternis albicollis*) and chicken reveals extensive fusions and fissions during karyotype evolution of accipitridae (Aves, Falconiformes). Chromosome Res, 2010; 18: 349–355.

87. de Oliveira, E. H. C.; Tagliarini M. M.; Santos, M. S.; O´Brien, P. C. M. & Ferguson-Smith, M. A. Chromosome painting in three species of buteoninae: a cytogenetic signature reinforces the monophyly of south american species. Plos One, 2013; 8(7), 5–10.

98. Nanda I; Karl E; Volobouev V; Griffin DK; Schartl M; Schmid M. Extensive gross genomic rearrangements between chicken and Old World vultures (Falconiformes: Accipitridae). Cytogenet Genome Res, 2006; 112:286-295.

109. Nishida C, Ishijima J, Ishishita S, Yamada K, Griffin DK, Yamazaki T, et al. Karyotype reorganization with conserved genomic compartmentalization in dot-shaped microchromosomes in the Japanese mountain hawk-eagle (*Nisaetus nipalensis orientalis*, Accipitridae). Cytogenet Genome Res, 2013;141:284–94.

110. Nie, W.; O´Brien, P. C. M.; Fu, B.; Wang, J.; Su, W.; He, K.; Bed´Hom, B. et al. Multidirectional chromosome painting substantiates the occurrence of extensive genomic reshuffling within Accipitriformes. BMC Evolutionary Biology, 2015; 15:205.

12. O'Connor, R. E.; Kiazin, L.; Skinner, B.; Fonseka, G.; Joseph, S.; Jennings, R. et al. Patterns of microchromosome organization remain highly conserved throughout avian evolution. Chromosoma, 2018; 128: 21–29.

13. Waters, P. D.; Patel, H. R.; Ruíz-Herrera, A.; Álvarez-González, L.; Lister, N. C.; Simakov, O.et al. Microchromosomes are building blocks of bird, reptile and mammal chromosomes. BioRxiv [Preprint] 2021 bioRxiv [Posted 2021 July 07]. Available from: https://www.biorxiv.org/content/10.1101/2021.07.06.451394v1
doi: 10.1101/2021.07.06.451394.

14. Furo, I.O.; Kretschmer, R.; O'Brien, P.C.M.; Pereira, J.; Garnero, A.D.V.; Gunski, R. et al. Chromosomal evolution in the phylogenetic context in Neotropical Psittacidae with emphasis on a species with high karyotypic reorganization (*Myiopsitta monachus*). Front. Genet. 2020, 11: 721.

15. Kretschmer, R.; de Souza, M.S.; Furo, I.d.O.; Romanov, M.N.; Gunski, R.J.; Garnero, A.d.V.; de Freitas, T.R.O.; de Oliveira, E.H.C.; O’Connor, R.E.; Griffin, D.K. Interspecies Chromosome Mapping in Caprimulgiformes, Piciformes, Suliformes, and Trogoniformes (Aves): Cytogenomic Insight into Microchromosome Organization and Karyotype Evolution in Birds. Cells, 2021, 10: 826.

16~~1~~. Crooijmans, R.; Vrebalov, J.; Dijkhof, R; Van Der Poel, J. & Groenen, M. Two-dimensional screening of the Wageningen chicken BAC library. Mammalian Genome, 2000; 11(5): 360-363.

17~~2~~. Lithgow, P. E.; O´Connor, R.; Smith, D.; Fonseka, G.; Al Mutery, A.; Rathje, C.; Frodsham, R et al. Novel tools for characterising inter and intra chromosomal rearrangements in avian microchromosomes. Chromosome Res, 2014; 22:85–97.

18. Rangel, T.F.L.V.; Diniz-Filho, J.A.F. Worldwide patterns in species richness of Falconiformes: Analytical null models, geometric constraints, and the mid-domain effect. Braz. J. Biol., 2004, 64: 299-308.

19. Degrandi T, M, Barcellos S, A, Costa A, L, Garnero A, D, V, Hass I, Gunski R, J: Introducing the Bird Chromosome Database: An Overview of Cytogenetic Studies in Birds. Cytogenet Genome Res 2020. doi: 10.1159/000507768~~Sick, H. Ornitologia Brasileira. 2ed. Rio de Janeiro, Nova Fronteira. 1997~~.

Formatted: English (United Kingdom)

Formatted: English (United States)

Formatted: English (United States)

Field Code Changed

Formatted: English (United States)

Formatted: English (United Kingdom)

Formatted: English (United Kingdom)

~~13. Sasaki, M. et al. A Feather Pulp Culture Technique for Avian Chromosomes, with Notes on the Chromosomes of the Peafowl and the Ostrich. Experientia, 1968; 24(12):1292-1293.~~

20~~14~~. Haring, E.; Kvaloy, K.; Gjershaug, J.; Rov, N. & Gamauf, A. Convergent evolution and paraphyly of the hawk-eagles of the genus *Spizaetus* (Aves, Accipitridae) – phylogenetic analyses based on mitochondrial markers. J Zool Syst Evol Res, 2007; 45(4): 353–365.

21~~15~~. Romanov, M. N.; Farré, M.; Lithgow, P. E.; Fowler, K. E.; Skinner, B. M.; O'connor, R.; Fonseka, G.; Backström, N.; Matsuda, Y.; Nishida, C et al. Reconstruction of gross avian genome structure, organization and evolution suggests that the chicken lineage most closely resembles the dinosaur avian ancestor. BMC Genomics, 2014; 15:1060.

22~~16~~. Zhang, G.; Li, C.; Li, Q.; Li, B.; Larkin, D. M.; Lee, C.; Storz, J. F. et al. Comparative genomics reveals insights into avian genome evolution and adaptation. Science, 2014; 346: 1311-1319.

2~~1~~3. Sasaki, M. et al. A Feather Pulp Culture Technique for Avian Chromosomes, with Notes on the Chromosomes of the Peafowl and the Ostrich. Experientia, 1968; 24(12):1292-1293.

Figure Legends

Figure 1- Metaphase (A) and karyotype (B) of ~~*Spizaetus*~~ *S. tyrannus* with diploid number 2n = 68, obtained with Giemsa conventional staining. The red arrows in (A) indicate the sex chromosomes. Scale bar: 5µm

Formatted: Font: 12 pt

Figure 2- Representative results of FISH experiments using ~~*Gallus*~~ *G. gallus* chromosome-specific probes corresponding to pairs GGA1 to GGA5 in ~~*Spizaetus*~~ *S. tyrannus* karyotype. Red and green signals represent probes labelled with Cy3 or FITC, respectively. Scale bar: 5µm

Figure 3- Representative results of hybridizations with some ~~*Gallus*~~ *G. gallus* BACs probes in the karyotype of ~~*Spizaetus*~~ *S.tyrannus*. (A1 and A1.1) Chicken BAC17 was the only one to hybridize on different chromosomes. Red signals represent probes labeled with Cy3, corresponding to the proximal region (p); Green signals represent probes labeled with FIT~~I~~C, corresponding to the distal region (d). Arrows indicate the signals. Scale bar: 5µm

Figure 4- Idiogram representing the homology between the ~~*Spizaetus*~~ *S. tyrannus* chromosomes and the macrochromosome chromosome-specific probes and microchromosomes BAC clones from ~~*Gallus*~~ *G. gallus*. Empty boxes mean no signal detected in those chromosomes with the set of probes used. BACs corresponding to pairs 20 and 22 (*) did not produced any detectable signals.

UNIVERSIDADE FEDERAL DO PARÁ
INSTITUTO DE CIÊNCIAS EXATAS E NATURAIS
FACULDADE DE CIÊNCIAS NATURAIS

RESPONSE TO REVIEWERS

Belém, September, 17, 2021.

Dear Reviewers,

We are thankful for the constructive reviews that we received; they certainly helped us

to improve the manuscript. Please find below our responses to each of your comments.

Please find below our responses to each of your comments.

Reviewer #1:

1- The work from Carvalho et al brings and interesting analysis in the in the Black-Hawk-Eagle (*Spizaetus tyrannus*) using BAC and WCP experiments, both derived from *Gallus gallus*. The results are very well represented in high-quality figures and the and brings new pieces of information for the big puzzle that avian karyotype evolution represents. Below I indicate some points to be adjusted and put special attention to one suggestion for discussion: The work would profit too much if the analyses were done under a phylogenetic context. An updated phylogeny with the special focus on the phylogenetic position and relation between Chicken and *Spizaetus* would bring more light to the general results found.

A- From the cytotaxonomic point of view, *Gallus gallus* has a basal karyotype, very similar to the avian putative ancestor. As this species is an important biological model, and because it also retained a plesiomorphic karyotype, GGA probes are used as a standard for chromosomal studies in birds. However, except for the use

of other probes, which could reveal intrachromosomal rearrangements, a phylogenetic analysis of GGA and birds of prey is not very informative, except as outgroup

Abstract

2- I missed some introduction about BIRDs cytogenetic in the beginning of Abstract. The authors go too direct about Accipitriformes and most readers do not have such phylogenetic information to which group we are dealing with.

A- We agree, and added an introduction to the abstract "*Although most birds show karyotypes with diploid number around 2n=80, with few macrochromosomes and many microchromosomes pairs, some groups, such as the Accipitriformes, are characterized by a large karyotypic reorganization, which resulted in complements with low diploid numbers, and a smaller number of microchromosomal pairs when compared to other birds*"

3- Why are the authors aiming to investigate homologies with chicken?

A- Because this species is an important biological model, and because it also retained a plesiomorphic karyotype, GGA probes are used as a standard for chromosomal studies in birds. We added a small explanation in the introduction "*In general, studies focusing on chromosome evolution in birds are based in comparative chromosome painting using chicken whole chromosome probes (Gallus gallus – GGA, 2n=78), due to the similarity of the karyotype of this species with the PAK [5].*"

4- Abstract also brings some previous data on NNI that were not originated from this work. I suggest removing.

A- We removed it

5- The terms STY and NNI are used without any previous information/explanation about, causing misunderstandings.

A- We corrected it

6- A final conclusion is missing in the abstract.

A- We added a conclusion in the abstract: "*Comparison with hybridization pattern of the Japanese-Mountain-Eagle, the only Aquilinae analyzed by comparative chromosome painting previously, did not reveal any synapomorphy that could represent a chromosome signature to this subfamily. Therefore, conclusions about karyotype evolution in Aquilinae require additional painting studies*".

Introduction

7- Page3 line3: Present an unusual (delete with)

A- We deleted it.

8- Page 3 line 12: The accurate identification of the chromosomal pairs involved.… if we aim to identify synapomophies….

A- We corrected it

9- Introduction miss a clear motivation for the study.

A- We improved it: "*Among them, the subfamily Aquilinae includes medium and large species, distributed globally, usually known as booted eagle. Usually, ten genera are found within Aquilinae. Cytogenetically, the only information concerning Aquilinae is the definition of the diploid number of six species (four genera), ranging from 2n=66 to 82 [19]".*

10- Why using GGA probes?

A- Because this species is an important biological model, and because it also retained a plesiomorphic karyotype, GGA probes are used as a standard for chromosomal studies in birds. We added a small explanation in the introduction.

11- I missed some info (Figure would be better) about the phylogenetic position and relation between Chicken and *Spizaetus*.

A- The authors agree that for this study are not necessary introduce some information about the phylogenetic position between *Gallus gallus* and *S. tyrannus*, however short information of phylogenetic relationship of the *S.tyrannus* within the Aquilinae Subfamily were added in the introduction aiming improve the reading of this paper.

12- Moreover, inform here as well thar STY corresponds to *Spizaetus tyrannus* on its first mention.

A- This was corrected.

Results

13- Subtitle Karyotypes before the first paragraph.

A- We added it.

Figures

14- Figure 3: I did not understand why using the metaphases present in right column? The signals and chromosomal morphology are clearly visible in the FISH images. Scale bars are missing in all figures.

A- We removed the right column and added the scale bars in all figures.

Reviewer #2:

In this study, the authors used comparative chromosome painting to reveal homology of chromosomal segments between *Spizaetus tyrannus* (the Black-Hawk-Eagle) and *Gallus gallus*. The study is conceptually and methodically motivated. The karyotype of *S. tyrannus* was previously studied only using conventional chromosome staining technique that showed 2n=68 (32m/sm+8st+18a+8m+ZW), and this happened for the first time that the karyotype of this bird of prey was studied by comparative chromosome painting. As a result, the study provides a novel insight into the cytogenetics of the Black-Hawk-Eagle. Both whole chromosome-specific *G. gallus* probes of the 1st-10th pairs and chromosome-specific *G. gallus* BAC probes from 11 pairs of microchromosomes were used. The study evidenced 29 evolutionary fission-fusion events that happened in the evolution of *S. tyrannus* and identified the particular chromosome pairs in the referenced *G. gallus*

karyotype, which have undergone restructuring or have remained unchanged. Another sufficient result concerns the comparison between *S. tyrannus* and *Nisaetus nipalensis orientalis*, which is its only close relative studied so far by comparative chromosome painting. The comparison showed both similarities and differences between the species. The MS is well illustrated by tables and pictures of good quality. In general, the work is interesting and deserves publication in the journal. However, there are some shortcomings in the work. Main disadvantages are (1) almost complete absence of basic data on the karyotypes of the discussed species, which makes it difficult to adequately assess the results obtained, and (2) ignorance of the taxonomic component.

A- We try to improve it.

All other comments that I have are mainly suggestions for improving the article (unfortunately, there is no line numbering in the MS, which makes it difficult for the reviewer to work).

1-Running title. It is too long, should be shorter, e.g., "Comparative Chromosome Painting in *Spizaetus tyrannus* and *Gallus gallus*".

A- W corrected it.

2. Abstract. Please, decipher GGA, should be *Gallus gallus* (GGA), as done above for *Spizaetus tyrannus* (STY)

A- We corrected it.

Introduction.

3- Page 3. Paragraph 1. Please, provide a putative avian ancestral karyotype, with 2n and the number of microchromosomes.

A- We provided this information in the introduction of the paper.

4- Page 3. Paragraph 1. Here (or further, in Discussion), provide the *G. gallus* karyotype to make further reasoning clearer.

A- We did it.

5- Page 3. Paragraph 3. Unify way of citing – in other places you use numbering.

A- We corrected it.

6- Page 4. Paragraph 4. Please, specify the species studied, *Spizaetus tyrannus*

A- We corrected it.

Discussion.

7- Page 6, Paragraph 1. Please, expand the statement “We report slight differences in chromosome morphology….” by clarifying what Tagliarini et al. (2007) have reported. Describe the differences, give for comparison one and the other karyotypes (otherwise, you only have a declaration).

A- In order to clarify the difference in chromosome morphology described between this study and the Tagliarini et al., (2007), we made a comparative table that was introduce in the results.

8- Page 7. Paragraph 3. The Japanese mountain hawk-eagle *Nisaetus nipalensis orientalis* (Temminck & Schlegel, 1844), not the Hodgson's hawk-eagle *Nisaetus nipalensis* Hodgson, 1836.

A- We corrected it.

9- Other remarks can be found in the MS attached.

A- We followed all the instructions of attached.

Reviewer #3:

This study provides a cytogenetic mapping of the Black-Hawk-Eagle (*Spizaetus tyrannus*) using whole-chromosome paints and BAC probes of *Gallus gallus*. Using this

cytogenetic mapping, the authors investigate the chromosome homologies between *Spizaetus tyrannus* and *Gallus gallus*. Overall the manuscript presents an advance in cytogenetic of Accipitriformes. The manuscript is written in understandable English, but some procedures require more details. Please see below my comments that could help to further enhance the quality of the study.

2) Abstract: "chicken (*Gallus gallus* – GGA)".

A- We corrected it.

3) Introduction and discussion: The manuscript is written in a manner suitable for a more specialized journal such as Cytogenetic and Genome Research or Comparative Cytogenetics, but not ideally for a general journal such as PlosOne. The introduction and discussion are rather limited to the focus of the analyses. This is a shame as I think the authors did a good job to gather nice cytogenetic results. Thus, the authors should consider redrafting these sections on a broad context.

The introduction could be more informative about the study as a whole. For example, the authors can give information about the contribution of this kind of work to karyotypic evolution and chromosomal organization of Accipitriformes. In this line, the last paragraph of introduction can be better written showing the importance of this study, and not merely comment "This study presents the cytogenetic mapping of a species…". Moreover, other intriguing topic is about the origin of microchromosomes. And maybe the authors could comment a little bit about this topic in the introduction and also discussion. Below is a reference related to this topic.

- Waters, P. D., Patel, H. R., Ruíz-Herrera, A., Álvarez-González, L., Lister, N. C., Simakov, O., ... & Graves, J. A. M. (2021). Microchromosomes are building blocks of bird, reptile and mammal chromosomes. bioRxiv.

A- The introduction was rewritten and several new information were added to improve the understanding of the manuscript. Also, some information from the reference recommended by the reviewer were considerate in this topic.

4) Results, paragraph 1: The authors commented that they detected “four pairs of microchromosomes” but they indicated five (29, 30, 31, 32, and 33). Considering the Figure 1B the pair 29 apparently is not a microchromosome.

A- We reviewed the Karyotype of *S.tyrannus* and considered only four pairs of microchromosomes (Pairs: 30, 31, 32 and 33). Also, we corrected it throughout of the text.

5) Results, paragraph 2: “The most extreme examples are the fission of GGA1 into six pairs in STY, and GGA3 into three distinct pairs”. It seems that for GGA3 are four pairs (13, 16, 19, and 20) (Figure 4), right?

A- The reviewer is right. The GGA3 probe correspond to four pairs (13,16,19 and 20).
We changed it.

6) Discussion, paragraph 3: “…karyotype of STY when compared to *Gallus*”; “…karyotype of STY when compared to *Gallus gallus*”.

A- We corrected it.

7) Discussion, paragraph 4: “…microchromosomes (STY4, 7 and 9; NNI2, 4 and 9), none of them”; “microchromosomes (STY: pairs 4, 7 and 9; NNI: pairs 2, 4 and 9), none of them”.

“…with microchromosomes (GGA: pairs 18, 19, 24 and 25)”.

A- We corrected it.

8) Discussion, paragraph 5: “These results show that they are morphologically similar species that until the last decade were part of the same genus [14].”. I think the authors results did not show an association of chromosomal data with morphological similarity and this should be corrected.

A- We removed it.

9) Conclusion: "…of *S.tyrannus* should be…"; "…of *S. tyrannus* should be…".

A- We corrected it.

10) Methods, paragraph 1: What the name and country of the Zoos?

A- The information was added in the Methods.

11) Methods, paragraph 2: "…and labelled directly by FTIC"; "and labelled directly by fluorescein isothiocyanate (FITC)".

A- We changed it.

12) Methods: the results of this study are based on FISH with GGA probes. However, FISH procedure is not sufficiently described. I would appreciate if at least main/important steps of the FISH procedure are described. This would also avoid any doubt about the accuracy of the results.

A- We provided the main steps of the FISH procedure.

13) Figure 3: "FITC".

A- We corrected it.

14) Please, give a scale bar information for all figures. It is very important, specially considering the presence of macrochromosomes and microchromosomes.

A- We added the scale bar in all figures.
