# Supplementary material for: Comparative chromosome painting in Spizaetus tyrannus and Gallus gallus with the use of macro- and microchromosome probes
Source: PLoS One. 2021 Nov 18;16(11):e0259905. doi: 10.1371/journal.pone.0259905 (PMC8601422; doi:10.1371/journal.pone.0259905)
Supplement: Attachment — Submitted filename: Carvalho et al-Response to the reviewers -2-Plos One.docx [file pone.0259905.s004.docx]

**UNIVERSIDADE FEDERAL DO PARÁ**

**INSTITUTO DE CIÊNCIAS EXATAS E NATURAIS**

**FACULDADE DE CIÊNCIAS NATURAIS**

RESPONSE TO REVIEWERS

Belém, October, 27, 2021.

Dear Editor,. Dear reviewers

We are thankful for the constructive reviews that we received. Please find below our responses to each of the comments sent by the reviewers.

Sincerely,

The Authors.

**Reviewer #2:** The manuscript has been extensively revised. Generally, all my comments were addressed and I am satisfied with the revision. However, minor typos remain (listed below and marked in the attached PDF), which can be corrected while editorial processing and preparing the manuscript for publication.

**Answer: Thank you very much for the positive feedback, we accepted all the suggestion in the PDF file.**

Title

1. Comparative Chromosome Painting in the Black-Hawk-Eagle (Spizaetus tyrannus) and Gallus gallus with the use of Macro and Microchromosome Probes. It is better to use only Latin names in both cases, e.g. Comparative Chromosome Painting in Spizaetus tyrannus and Gallus gallus with the use of Macro-and Microchromosome Probes. Then, Macro-and Microchromosome Probes should be written with a hyphen.

**Answer: Thank you, we accepted the suggestion.**

Abstract

1. Here and elsewhere (Introduction, Results), should not be duplicated diploid number and 2n, as for example in the case “diploid number around 2n=80”. Should be either diploid number around 80 or 2n around 80

**Answer: We corrected it.**

2. The family Accipitridae contains 14 subfamilies, not only Aquilinae, then, the phrase “the Accipitridae family is the most diverse and includes the subfamily Aquilinae…” needs to be revised (see my suggestion in the PDF text).

**Answer: We corrected it.**

Results

1. Table 3. Please, explain in the title the abbreviation “q”.

**Answer: We explained it.**

**Reviewer #3:** Carvalho et al. 2021 – Resubmission “Comparative Chromosome Painting in the Black-Hawk-Eagle (Spizaetus tyrannus) and Gallus gallus with the use of Macro and Microchromosome Probes”. This is a re-review of the manuscript for PlosOne. The new version of the manuscript is improved, and I do think the results overall are publishable. I only have a few suggestions that can be considered during the production process.

**Answer: Thank you very much for the positive feedback.**

- Please alphabetic order: “…In fact, rearrangements involving microchromosomes were detected in few orders: Psittaciformes, Cuculiformes, Suliformes, Caprimulgiformes and the Accipitriformes [13-15].”

**Answer: We corrected it.**

-“Gallus gallus probes used in the fluorescent in situ hybridization (FISH) experiments produced…”.

**Answer: We corrected it.**
